# Enhancing Composite Toughness Through Hierarchical Interphase Formation

**DOI:** 10.1002/advs.202305642

**Published:** 2023-12-25

**Authors:** Sumit Gupta, Tanvir Sohail, Marti Checa, Sargun S. Rohewal, Michael D. Toomey, Nihal Kanbargi, Joshua T. Damron, Liam Collins, Logan T. Kearney, Amit K. Naskar, Christopher C. Bowland

**Affiliations:** ^1^ Carbon and Composites Group Chemical Sciences Division Oak Ridge National Laboratory Oak Ridge TN 37830 USA; ^2^ Advanced Computing for Chemistry and Materials Group National Center for Computational Sciences Oak Ridge National Laboratory Oak Ridge TN 37830 USA; ^3^ Functional Atomic Force Microscope Group Center for Nanophase Materials Sciences Oak Ridge National Laboratory Oak Ridge TN 37830 USA

**Keywords:** fiber‐matrix adhesion, fiber‐matrix interphase, fiber‐reinforced composites, hierarchical architecture, nanofiber scaffold

## Abstract

High strength and ductility are highly desired in fiber‐reinforced composites, yet achieving both simultaneously remains elusive. A hierarchical architecture is developed utilizing high aspect ratio chemically transformable thermoplastic nanofibers that form covalent bonding with the matrix to toughen the fiber‐matrix interphase. The nanoscale fibers are electrospun on the micrometer‐scale reinforcing carbon fiber, creating a physically intertwined, randomly oriented scaffold. Unlike conventional covalent bonding of matrix molecules with reinforcing fibers, here, the nanofiber scaffold is utilized ‒ interacting non‐covalently with core fiber but bridging covalently with polymer matrix ‒ to create a high volume fraction of immobilized matrix or interphase around core reinforcing elements. This mechanism enables efficient fiber‐matrix stress transfer and enhances composite toughness. Molecular dynamics simulation reveals enhancement of the fiber‐matrix adhesion facilitated by nanofiber‐aided hierarchical bonding with the matrix. The elastic modulus contours of interphase regions obtained from atomic force microscopy clearly indicate the formation of stiffer interphase. These nanoengineered composites exhibit a ≈60% and ≈100% improved in‐plane shear strength and toughness, respectively. This approach opens a new avenue for manufacturing toughened high‐performance composites.

## Graphical abstract

High strength and ductility are highly desired in fiber‐reinforced composites, yet achieving both simultaneously remains elusive. A hierarchical architecture is developed utilizing high aspect ratio chemically transformable thermoplastic nanofibers that form covalent bonding with the matrix to toughen the fiber‐matrix interphase. This approach is practical yet straightforward and can potentially produce composites with superior fiber‐matrix interfacial properties.

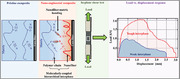

## 1 Introduction

Fiber‐reinforced polymer composites (FRPCs) are ubiquitous in aerospace, automotive, and renewable energy industries primarily due to their excellent specific strength and resilience in aggressive environments.^[^ ^1^ ^]^ FRPCs are generally fabricated by infusing continuous or chopped inorganic and organic fibers with thermosetting or thermoplastic polymers. However, due to their inherent heterogeneity, conventional FRPCs suffer from poor fiber‐matrix interfacial bonding, hindering the fiber‐matrix load transfer.^[^ ^2^ ^]^ Since the fiber‐matrix interphase dictates the mechanical properties of the bulk composites, significant research effort has been made to enhance these interphase properties.^[^ ^2^, ^3^, ^4^ ^]^ A tough interphase that is simultaneously strong and ductile is essential to guarantee the FRPCs’ improved structural performance. According to Wegst and Ashby, such demands are often challenging due to the inverse correlation between strength and ductility.^[^ ^5^ ^]^

With the recent advances in polymer and processing sciences, including the incorporation of nanomaterials, significant enhancements in fiber‐matrix adhesion have been achieved. A key component in this pursuit is the use of fiber sizing, a thin polymeric coating applied to the fiber surface that serves as a protective layer and facilitates ease of handling while promoting fiber‐matrix adhesion.^[^ ^6^, ^7^ ^]^ For example, typical glass fiber sizings contain an organosilane compound that forms a polysiloxane network through hydrolysis and subsequent polycondensation.^[^ ^8^, ^9^ ^]^ Abundant ─OH on the glass fiber surface chemically bonds with this hydrolyzed organosilane, resulting in a semi‐interpenetrating network implanted within the polymer matrix. Such networks significantly improve the fiber‐matrix interaction, resulting in a >50% increment in interfacial shear strength (IFSS).^[^ ^10^ ^]^ Thermoplastic poly(phthalazinone ether ketone) as carbon fiber sizing forms epoxy resin‐compatible polar groups on the fiber surface, producing a composite with ≈15% higher IFSS.^[^ ^11^ ^]^ This paper also reported a ≈56% gain in fracture toughness attributed to the interfacial bond strength increasing via sizing. Atkins et al.^[^ ^12^ ^]^ applied fiber sizing in a periodic fashion, thus intentionally creating high and low‐shear stress regions on the fiber surfaces during mechanical loading. While the sized fiber portions ensure strength, the weak spots could blunt the crack growth, resulting in fourfold enrichment in a composite's fracture toughness. Polymer grafting^[^ ^13^, ^14^ ^]^ and plasma polymerization^[^ ^15^, ^16^, ^17^, ^18^ ^]^ were recently investigated as feasible options for efficient integration of suitable sizing on the fiber surfaces. Deng et al. utilized carbon fiber's chemical grafting with a soft hydroxyl‐terminated diblock copolymer poly (*n*‐butylacrylate)‐b‐poly (glycidyl methacrylate) (OH‐PnBA‐b‐PGMA) that displays 30% better energy absorbing property than its pristine counterpart.^[^ ^13^ ^]^ Ore´fice et al.^[^ ^14^ ^]^ reported that a chemically modified poly (aryl ether sulfone) macromolecule, when grafted with the bioactive glass particles, shows a 20% improvement in interfacial strength. Furthermore, the macromolecule's hyperbranched architecture helps to absorb ≈100% more energy during interfacial failure.

The effect of fiber sizing on the interfacial strength can be further magnified by enhancing the sizing materials with nanofillers. Nanomaterials typically possess dimensions at least one order of magnitude smaller than the core fibers. Such deliberate arrangement of materials with ordered dimensions establishes a hierarchical structure, which has been extensively investigated to enhance the interphase between fibers and matrices, thereby promoting superior strength. Here, they exaggerate the fiber surface roughness while increasing the average stiffness of the interphase, thereby offering a better interfacial property. For instance, carbon nanotubes (CNTs)^[^ ^19^ ^]^ and graphene^[^ ^20^ ^]^ were used as reinforcing elements with the fiber sizing to attain a superior IFSS (>25% on average). Our recent work also demonstrated that ceramic nanoparticles (TiO_2_, BaTiO_3_, and SiC) could be efficiently integrated at the fiber‐matrix interphases, bettering the FRPCs’ interlaminar shear strength.^[^ ^21^, ^22^, ^23^, ^24^, ^25^ ^]^ Although fiber sizing is a straightforward way to boost the fiber‐matrix interaction, selecting compatible yet universal sizing elements is crucial.^[^ ^26^, ^27^ ^]^

Alternatively, the structural hierarchy could be created by directly dispersing the nanofillers into the polymer matrix and used in composite casting for IFSS improvement. Hossain et al. demonstrated that amine‐functionalized CNTs, incorporated within resin via sonication, enhance the interlaminar shear strength by ≈22%.^[^ ^28^ ^]^ A magnified yet similar trend in IFSS (≈60% improvement) was observed by Pedrazzoli et al., where the matrix was engineered with exfoliated graphite nanoplatelets in glass fiber‐reinforced composites.^[^ ^29^ ^]^ In addition, the resulting composite displays an improved storage modulus and viscoelastic behavior as an outcome of improved fiber‐matrix interactions and immobilization of the polymer chains caused by the nanomaterials.^[^ ^29^ ^]^ Despite the simplicity of the nanomaterials‐based matrix enhancement method, uniform yet high‐volume nanomaterials dispersion can only be achieved through advanced dispersion techniques. High‐volume nanomaterials agglomerate reduces the bulk mechanical properties of the composites.^[^ ^30^ ^]^

Instead of mixing nanomaterials with the sizing or the bulk polymer matrix, they could be anchored to the fibers through chemical bonding to realize a more efficient fiber‐matrix load transfer ─OH on glass fibers, for instance, can be converted into ─NH_2_ that can covalently bond with the ─COOH available in graphene oxides and CNTs via amide coupling, imparting a ≈50% augmented IFSS.^[^ ^31^ ^]^ CNTs can also be directly grown on the fiber surface through chemical vapor deposition (CVD).^[^ ^32^ ^]^ In this two‐step process, the carbon fiber surface is first coated with a suitable catalyst, followed by CNT grown within a reactor using a hydrocarbon source (600–800 °C). The hierarchical architecture of CVD‐grown CNTs amplifies the fiber surface area while offering mechanical interlocking and enhanced interphase stiffness resulting in ≈60% higher IFSS^[^ ^33^ ^]^ and toughness.^[^ ^34^ ^]^ More recently, ZnO nanowires have been explored as a viable option to be grown on Aramid fiber surfaces at a comparatively lower temperature (150 °C). First, aramid fiber amide bonds are cleaved by NaOH, and its Na^+^ is exchanged by H^+^, forming ─COOH on the fiber surface. These ─COOH groups interact with Zn^2+^, producing a coordination complex offering a ≈50% rise in IFSS.^[^ ^35^, ^36^ ^]^

**Figure 1:**
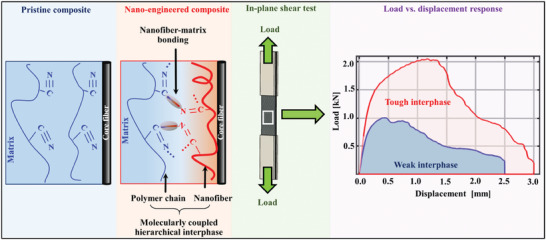
A schematic illustration of fiber‐matrix interphase with PAN nanofibers (≈0.35‐µm‐diameter) deposited on the ≈5‐µm‐diameter carbon fiber surface creating a hierarchical architecture covalently bonded with ABS matrix. An in‐plane shear strength test was performed to characterize the composites’ interfacial properties. While the representative load‐displacement curve of pristine composites (i.e., the composites without any hierarchical nanofibers) exhibit weak behavior (blue curve), the nanofiber‐enhanced composites are tougher with higher work of fiber‐matrix debonding (red curve). The C─N bond formed between PAN and ABS resulted in a co‐continuous interphase between the core fiber and the matrix, resulting in stronger and tougher composites.

The above discussion about hierarchical structures grafting on the core fiber surface suggests that hierarchical design‐enabled tough composites could also be achieved from the matrix side, i.e., by bonding them physically with fibers and chemically with the polymer matrix. To the best of our knowledge, this is the first report where we tailor the fiber‐matrix interfacial interaction by designing a hierarchical structure consisting of a nanofiber scaffold that is immiscible but molecularly coupled with the polymer matrix. Such chemomechanical scaffolding creates a strong biphasic component, thus enhancing the bulk composites’ mechanical performance. For the demonstration, poly(acrylonitrile‐butadiene‐styrene) (ABS), polyacrylonitrile (PAN), and carbon fiber were selected as the polymer matrix, the nanofiber scaffold, and the core fiber, respectively. The carbon fiber possesses a diameter that is one order of magnitude larger than that of electrospun nanofibers, thereby establishing a hierarchical network at the core fiber‐matrix interphase. PAN and ABS have compatible nitrile groups that can aid the desired hierarchical nanostructures‐matrix molecular coupling when subjected to suitable thermal stimuli. Electrospinning was used as a scalable and low‐cost method to integrate the high aspect ratio PAN nanofibers at the fiber‐matrix interphase.^[^ ^37^, ^38^, ^39^ ^]^ PAN‐ABS covalent bonding in the absence of carbon fiber was comprehensively studied through rheology characterization, mechanical testing, solubility testing, Fourier Transform Infrared (FTIR) spectroscopy, and solid‐state Nuclear Magnetic Resonance (NMR) spectroscopy. PAN nanofibers, when introduced at an FRPC's core fiber‐matrix interphase and molecularly coupled with the matrix, induce a stiffness gradient within the interphase, thus offering a better fiber‐matrix load‐transferring pathway. The nanoengineered fiber‐matrix interphase's elastic modulus was characterized via atomic force microscopy (AFM). A molecular dynamics (MD)‐based computational study explained the improved adhesion to the carbon fiber as a function of increasing PAN‐ABS covalent bonding, thereby providing further evidence of superior interfacial strength. Finally, the in‐plane shear strength test of the PAN‐enhanced FRPCs confirmed significant mechanical improvements resulting from the tough interphase obtained via hierarchical nanofiber‐matrix interfacial bonding (**Figure** **1**). This establishes an effective route to achieve a tough fiber‐matrix interphase that can be utilized for the design of future FRPCs with chemically different constituents.

## 2 Results and Discussion

**Figure 2:**
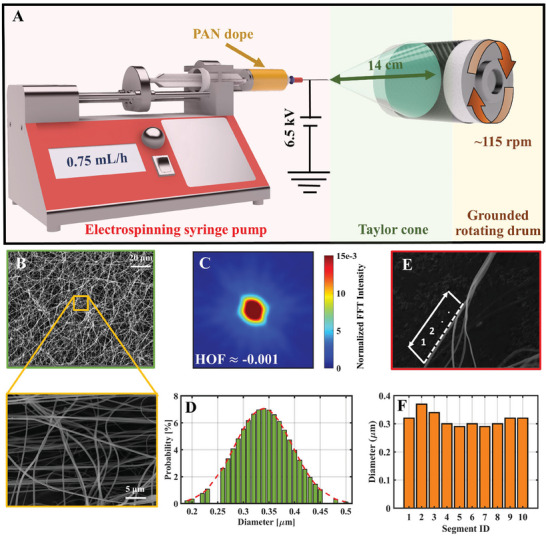
Electrospun PAN nanofibers’ morphology characterization. A) Electrospinning setup and the parameters used in PAN nanofiber electrospinning deposition. B) SEM image of the electrospun PAN nanofibers at different magnifications confirms their random yet uniform deposition. C) A normalized FFT plot of the electrospun PAN fibers’ SEM image is shown. D) PAN nanofiber diameter distribution is estimated. E) The diameter of the electrospun PAN nanofiber along its axis is measured at ten equidistantly spaced points, and F) its variation is plotted.

For electrospun PAN nanofibers’ morphological characterization, we deposited them on aluminum foil via electrospinning, schematically represented in **Figure** **2**A. Their morphology was observed through scanning electron microscope (SEM) imaging that demonstrates a randomly oriented well‐dispersed PAN fiber network (Figure 2B).

It is well‐known that fibrous materials generally have superior mechanical properties along their axis due to molecular alignment.^[^ ^40^ ^]^ Therefore, it's worth seeing if the electrospinning parameters used for PAN nanofibers’ deposition (Figure 2A) oriented them along any specific direction. The fast Fourier transformation (FFT) intensity plot of Figure 2B (inset) shows that the PAN nanofibers’ pixels are distributed in an asymmetric circular shape (Figure 2C), indicating their random orientation. We calculated Herman's Orientation Factor (HOF) of this FFT intensity plot for quantitative analysis.^[^ ^41^ ^]^ More details about the HOF calculation can be found in the Supplementary Materials. HOF's close‐to‐zero magnitude (≈−0.001) validates that our electrospinning method was able to produce a randomly oriented network of PAN nanofibers. Thus, their alignment effect will have a minimum contribution toward the composites’ strength enhancement. The adoption of a rotating drum approache effectively reduced the potential for introducing coating variations that might result from electrospinning on a stationary unidirectional carbon fiber sheet. This, in turn, provides a higher level of consistency necessary for subsequent mechanical testing of the composite laminates fabricated using these PAN nanofiber‐coated unidirectional carbon fiber sheets.

Our analysis further shows that PAN nanofibers have an average diameter of ≈ 0.35 µm (Figure 2D). The SEM image of PAN fiber (Figure 2E), when further processed through a customized image processing algorithm, reveals a negligible along‐axis standard deviation (≈ 0.025 µm) in fiber diameter (Figure 2F).

### 2.1 PAN‐ABS Covalent Bonding Validation

**Figure 3:**
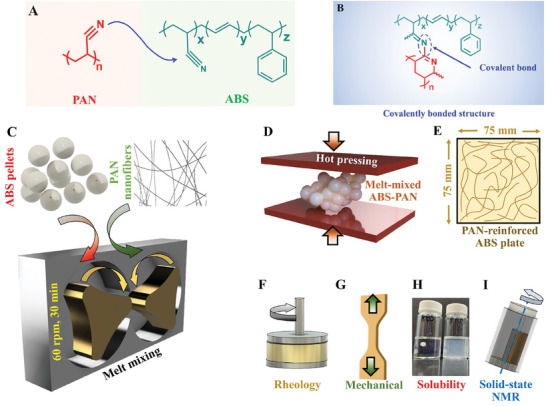
PAN‐ABS composites were used for their crosslinking validation. A) PAN and ABS's chemical structures are shown. B) presents the covalently bonded PAN‐ABS molecule. C) ABS pellets and electrospun PAN fibers were melt‐mixed within a shear mixer at 150 °C. D) Shear‐mixed PAN‐ABS was hot‐pressed at different temperatures under constant pressure to obtain E) PAN‐ABS composite plates. F) rheological characterization, G) uniaxial tensile test, H) solubility test, and I) solid‐state NMR spectroscopy were performed to validate the PAN‐ABS chemical bonding.

Initial validation of PAN‐ABS covalent bonding was performed prior to leveraging the concept for strengthening the fiber‐matrix interphase in FRPCs. PAN (molecular formula [C_3_H_3_N]*_n_*) (**Figure** **3**A), when subjected to heat treatment, produces thermally stable carbon fiber with well‐oriented molecular structures.^[^ ^42^ ^]^ In short, during thermal processing, the PAN fibers first undergo stabilization at a relatively low temperature (200–300 °C)^[^ ^43^ ^]^ with a conversion of PAN's C≡N into C═N, resulting in an infusible ladder polymer through cyclization (Figure S1, Supporting Information). This thermally induced fusion reaction of acrylonitrile moieties has been well characterized due to the important role they play as intermediates in the formation of carbon fiber.^[^ ^44^ ^]^ Acrylonitrile‐containing copolymers could be conventionally polymerized through free radical polymerization, which results in atactic expression of side group stereochemistry. The combination of segmental rigidity and lack of steric order in PAN yields a disordered helical arrangement of the chains with frustrated packing arrangements.^[^ ^45^, ^46^ ^]^ The cyclization of PAN can occur through inter‐ or intra‐molecular reactions.^[^ ^47^, ^48^ ^]^ In the conditioning of acrylic fibers for conversion to carbon fiber, intramolecular bonding between the nearest neighbors is preferred to favor the formation of a more ordered turbostratic carbon phase. In contrast, here, we utilize interchain reactions as covalent bonding between the periphery of the electrospun PAN nanofibers and the nitrile‐rich region of the ABS terpolymer to chemically couple the two phases and toughen the composite. More details about the other chemical steps and related PAN fiber chemistry can be found elsewhere.^[^ ^49^, ^50^, ^51^ ^]^

ABS (molecular formula [(C_8_H_8_)*_x_* ·​(C_4_H_6_)*_y_* ·​(C_3_H_3_N)*_z_*]*_n_*) (Figure 3A) is one of the most widely used thermoplastic polymers primarily known for its impact resistance, toughness, and rigidity.^[^ ^52^, ^53^ ^]^ According to our previous research, the thermal processing window of ABS (≈250 °C) ^[^ ^54^ ^]^ is congruent with the PAN's thermal window for its initial stabilization. We hypothesize that within this common temperature window, PAN and ABS could form covalent bonding without any modifications of the ABS's processing parameters. ABS and PANs’ nitrile constituents could be converted to C═N, similar to PAN's stabilization process described in Figure S1 (Supporting Information). Thereafter, C═N from ABS and PAN could react with each other, forming intermolecular C─N. The expected chemical reaction and the chemically bonded PAN‐ABS structure are shown in Figure 3B. It should be mentioned that several bonding modes can occur between acrylonitrile‐bearing molecules. However, the temperatures used in this study are in the range of previously reported crosslink formation through intermolecular addition.^[^ ^48^, ^55^ ^]^

Electrospun PAN fibers (≈ 0.01 wt.%) were shear‐mixed with ABS (Figure 3C), and hot‐pressed (Figure 3D), resulting in a PAN‐ABS composite represented by (PAN‐ABS)_T_, with T being the press temperature. The aforementioned wt.% of PAN was selected, assuming that ≈30 s of electrospinning time used in this study could only integrate PAN nanofibers at such ultra‐low concentration in the bulk composites. The shear‐mixed specimens were hot‐pressed at different temperatures to obtain PAN‐ABS composite (Figure 3E) with various covalent bonding levels. We performed three indirect (solubility test (Figure 3F), rheology study (Figure 3G), and mechanical characterization (Figure 3H)) and two direct (FTIR and solid‐state NMR spectroscopy (Figure 3I)) tests to validate PAN‐ABS covalent bonding hypothesis.

**Figure 4:**
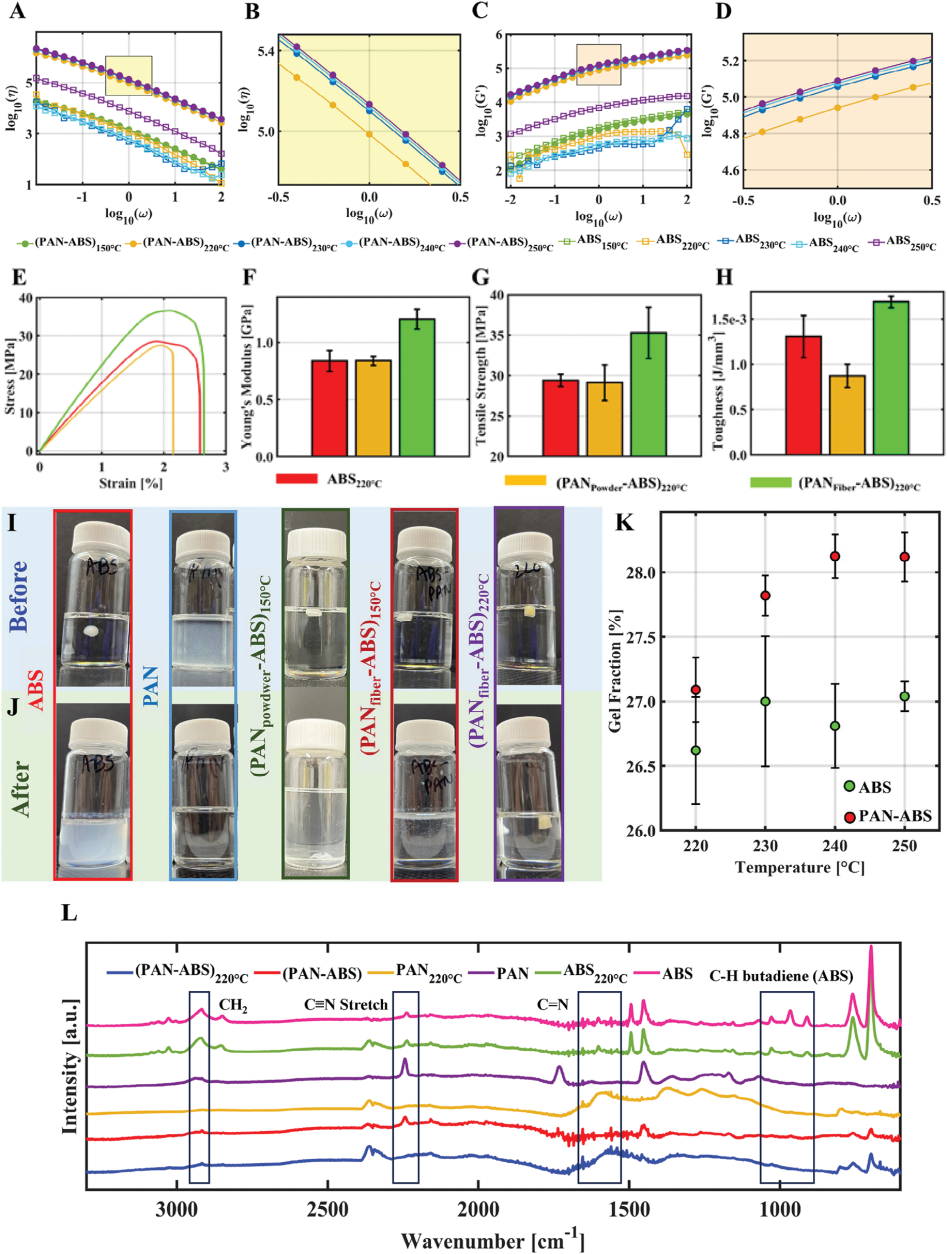
Indirect and direct test results to validate PAN‐ABS bonding. A–D) show the variation in *η* and *G’*, respectively, with angular frequency (*ω*) at different magnifications obtained from the rheology test. The units of *G’* and *η* are Pa and Pa s, respectively. B,D) are the zoomed‐in views of the data presented in (A) and (B), respectively. E) is a typical strain versus stress plot of the PAN‐ABS composites obtained from the mechanical testing. F–H) provide a summary of Young's modulus, tensile strength, and toughness of the PAN‐ABS composites, with six samples assessed within each set. Snapshots of different specimens I) before and J) after the solubility tests. K) summarizes the gel fraction of the PAN‐ABS composites. L) FTIR spectra of different (PAN‐ABS) specimens are overlaid.

Polymer chain scission and fusion during polymer–polymer covalent bonding results in microstructural transformation, thus influencing their viscoelastic responses. For example, escalation in rheological parameters (storage and loss modulus and viscosity) could be attributed to the increase in molecular weight due to polymer–polymer covalent bonding.^[^ ^56^ ^]^ Dynamic oscillation rheology is widely used as an indirect way for polymer–polymer bonding validation. The rheological responses during frequency sweep for PAN‐ABS specimens and neat ABS heat‐treated at different temperatures are shown in **Figure** **4**A,B. Compared to neat ABS, PAN‐ABS composites exhibit dramatic improvement in complex viscosity (*η*) and storage modulus (*G’*). We observed that the composites’ *η* gradually increases with their heat treatment temperature (Figure 4A,B). In fact, at the lower frequency, (PAN‐ABS)_220 °C_ has *η* two orders of magnitude higher than neat ABS. For example, at 0.01 rad s^−1^, *η* = 11778.6 Pa s and 1475,600 Pa s for neat ABS and (PAN‐ABS)_220 °C_, respectively. They further increased to 2233,700 Pa s for (PAN‐ABS)_250 °C_ specimens (Figure 4B). This observation could indicate more PAN‐ABS covalent bonding with increasing heat treatment temperature. Like *η*, the frequency‐dependent *G’* behaved in a similar fashion (Figure 4C,D). For instance, at 0.01 rad s^−1^, (PAN‐ABS)_220 °C_ has ten times higher *G’* than neat ABS. An upsurge in molecular weight due to PAN‐ABS covalent bonding could be responsible for such behavior of *G’*. We also found that neat ABS's rheological properties do not change with heat treatment temperature, consistent with the solubility test data below. As an ultralow concentration of PAN was maintained (0.01 wt.%), the reinforcing effect due to the self‐cyclization of PAN could be neglected. Hence, these observed trends in the rheological properties of the PAN‐ABS composites could be an indicator of PAN‐ABS covalent bonding.

Uniaxial tensile tests of PAN‐ABS composites were performed as another indirect way to prove the PAN‐ABS covalent bonding. Our hypothesis is that constituents of PAN‐ABS composite, when chemically bonded, would exhibit enhanced mechanical properties. In addition to casting PAN nanofiber‐based ABS composites, we also prepared and tested PAN powder‐based composites (PAN_powder_‐ABS) to characterize the effect of PAN's architecture on the bulk mechanical properties. The specimens’ typical strain versus stress responses are displayed in Figure 4E. In general, the composites’ strain versus stress response exhibits a linear regime followed by some yielding and failure. The composites’ Young's moduli (*E*) were determined by linear least square regression of the strain versus stress curves’ initial portion (from 0 to 0.01% strain regime) that shows (PAN‐ABS)_220 °C_ composites have an *E* ≈60% higher than neat ABS and (PAN_powder_‐ABS)_220 °C_ (Figure 4F). Additionally, (PAN‐ABS)_220 °C_ specimens have ≈20% and ≈50% higher tensile strength and fracture toughness, respectively (Figure 4G,H) than its pristine counterparts. Interestingly, (PAN_powder_‐ABS)_220 °C_ did not exhibit such improvement in mechanical properties. In fact, their *E* and tensile strength remains the same as the neat ABS, and the toughness is even compromised. While the PAN is still able to covalently bond with ABS, the powder form factor has less surface area; therefore, it can only offer a limited number of functional groups (C≡N) to facilitate PAN‐ABS bonding. On the other hand, the electrospun nanofiber scaffold has a high surface area and, thus, could provide more C≡N to be chemically bonded with the ABS. The molecular alignment in electrospun PAN nanofibers could also add some reinforcement effect; however, it would be negligible for their ultra‐low concentration (0.01 wt.%) within the bulk composite. The impact of the molecular alignment within PAN chains on the bulk composites will be explored in the future. We concluded that the long, entangled PAN and ABS chains were covalently bonded, resisting their unraveling under applied loading. Additionally, such bonded chains would be hard to separate, thus providing more tortuous pathways to crack propagation during failure. Such phenomena ultimately resulted in a composite with superior *E* and tensile strength. Moreover, the hierarchical network of the PAN fibers could absorb more energy prior to failure, thereby improving the toughness of the composites. This finding confirms the contribution of PAN fibers’ hierarchical architecture to enhance the composites’ mechanical properties. Shear mixing can lead to the separation, breakage, and shortening of PAN fibers, potentially impacting the mechanical properties of PAN‐ABS composites. Arash et al.^[^ ^57^ ^]^ have highlighted the benefits of maintaining the aspect ratio of reinforcing nanomaterials in improving the mechanical properties of the resulting composites. Thus, careful selection of shear mixing parameters (e.g., torque and mixing time) is crucial to prevent fiber fragmentation and mitigate its adverse effects on the mechanical properties of the final composites.

The covalently bonded PAN‐ABS composites were investigated via a solubility study.^[^ ^53^ ^]^ Here, specimens were added to a mixture of dimethyl sulfoxide (C_2_H_6_OS)/chloroform (CHCl_3_) (Figure 4I) as a common solvent (1:1, w/w, 48 h at 60 °C). Figure 4J shows that neat ABS, neat PAN, (PAN_powder_‐ABS)_220 °C_, and (PAN_fiber_‐ABS)_150 °C_ were completely dissolved. In contrast, (PAN‐ABS)_220 °C_ was not dissolved, indicating PAN‐ABS covalent bonding. These results further emphasize the significance of maintaining the fibrous structure of PAN within PAN‐ABS composites to achieve composites with enhanced mechanical properties.

The gel fractions^[^ ^58^ ^]^ of (PAN_fiber_‐ABS) composites prepared via various heat treatment temperatures are summarized in Figure 4K. We found that the gel fraction increases with the heat treatment temperature (220–240 °C) for (PAN_fiber_‐ABS) composites. A higher temperature could assist more PAN‐ABS bonding, causing an upsurge in the gel fraction. However, the gel fraction did not significantly increase after 240 °C, as indicated by the plateau in Figure 4K. At this temperature, a saturation point could have been reached where all the available C≡N on the PAN fiber surface became engaged with the ABS through covalent bonding. Although this result shows that PAN‐ABS bonding can be controlled by tuning the processing temperature, it also demonstrates that temperature above a threshold point may not necessarily help in more PAN‐ABS bonding. Overall, the solubility test results justify our hypothesis that PAN and ABS could form covalent bonding when subjected to a suitable temperature.

Spectroscopic evidence for the thermally induced changes in PAN and ABS as well as for PAN‐ABS composites were pursued with FTIR and are summarized in Figure 4L. The ABS specimens show alkene bonding modes in the low wavenumber region (900–940 cm^−1^), which deteriorate in intensity following oxidative exposure.^[^ ^59^, ^60^ ^]^ A methylene bending signature peak (2940 cm^−1^) can be seen for all as‐prepared samples prior to heat treatment. The PAN‐only specimen, following heat treatment, most clearly shows a decrease in the methylene intensity consistent with aromatization. The characteristic nitrile peak (2240 cm^−1^) is present in all neat samples and is nearly eliminated in the PAN_220 °C_ sample. This is likely due to the nature of the exothermic cascade resulting in a greater extent of reaction under identical thermal conditions in the PAN‐only specimen, which is moderated by the high amount of styrene and butadiene segments present in ABS. The loss of the intensity in the nitrile peak due to cyclization reactions corresponds to a new signal (≈1600 cm^−1^). These results are consistent with the solid‐state NMR results (Figure S2, Supporting Information) on PAN‐ABS covalent bonding validation.

Solid‐state NMR experiments were conducted as additional proof of PAN‐ABS covalent bonding in selective PAN‐ABS composites that underwent melt‐mixing at two temperatures, 150 and 220 °C. The (PAN‐ABS)_220 °C_ specimens exhibited increased rigidity, as evidenced by significant line broadening and the appearance of spinning sidebands in the ^13^C NMR results. This broadening affected both components, ruling out single‐phase‐only effects such as acrylonitrile cyclization. To further confirm these findings, ^1^H longitudinal relaxation experiments were performed on these specimens, as well as on pristine ABS and PAN. More details can be found in Supplementary Materials. Overall, the relaxation data suggests that in the (PAN‐ABS)_220 °C_ specimen, PAN and ABS are effectively mixing, displaying an intermediate relaxation time compared to the two individual samples. This observation substantiates our hypothesis that covalent bonding and cyclization between PAN and ABS molecules lead to phase rigidization.

In general, these indirect and direct tests certify that the hierarchical PAN fibers and ABS could be covalently bonded upon suitable heat treatment. Hence, the derived material system can be leveraged for nanoengineering the carbon fiber‐ABS matrix interphase for a tougher FRPC design.

### 2.2 Fiber‐Matrix Interphase Characterization in FRPCs

**Figure 5:**
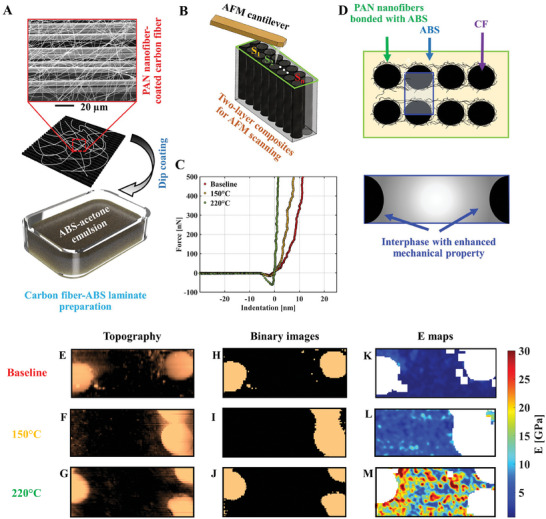
Microscale characterization of nano‐engineered fiber‐matrix interphase via AFM. A) Composites were prepared by dip‐coating PAN‐coated carbon fibers in ABS emulsion. The inset shows an SEM image of the PAN‐coated carbon fiber. B) Their surfaces transverse to the carbon fibers’ axes were polished and subjected to AFM scanning at various points (e.g., S_1_, S_2_,…, S_n_). Force‐indentation relationships at these probed locations were evaluated. C) showcases representative indentation vs. force curves at arbitrarily selected points from the fiber‐matrix interphase region for baseline, (PAN‐ABS)_150 °C_, and (PAN‐ABS)_220 °C_ specimens. D) Conceptual representation of anticipated interphase formation around the carbon fiber. E–G) are the AFM topography maps of the baseline, (PAN‐ABS)_150 °C_, and (PAN‐ABS)_220 °C_ specimens, respectively. In the corresponding binary images shown in H–J), the fiber areas are masked. *E* maps of the fiber‐matrix interphase region for K) baseline, L) (PAN‐ABS)_150 °C_, and M) (PAN‐ABS)_220 °C_ composites are shown.

Upon validating heat treatment‐enabled PAN‐ABS covalent bonding, we utilized the proposed nanofiber scaffold assembly to enlarge and improve the fiber‐matrix interphase region in FRPCs thus promoting a better fiber‐matrix load transfer. Here, we aim to characterize the stiffness distribution at the carbon fiber‐ABS matrix interphase via AFM. The same electrospinning parameters displayed in Figure 2A were utilized to deposit PAN nanofibers on unidirectional carbon fiber sheets. A representative SEM image of electrospun PAN fibers on carbon fiber is **Figure** **5**A (inset). We also estimated that ≈10.6% area of the carbon fiber sheet was coated by the PAN fibers (Figure S4A–C, Supporting Information) with ≈0.557 µm deposition thickness (Figure S4D, Supporting Information). We exploited these findings to estimate the wt.% of PAN fibers (≈0.013 or ≈0.026 wt.%, depending on whether only one or both sides of the carbon fiber sheet were subjected to electrospinning) in the final composites. The details of this procedure can be found in the Supplementary Materials. Despite the limitations in the assumptions of this procedure, these results prove that hierarchical PAN fibers with consistent morphology could be incorporated at an ultra‐low concentration within the composites’ fiber‐matrix interphase through electrospinning. FRPCs were prepared by stacking and hot pressing two layers of electrospun PAN nanofiber‐enhanced unidirectional carbon fiber sheets dip‐coated in acetone‐ABS emulsion (Figure 5A). The polished top surface of the specimen was subjected to contact‐mode AFM scanning (Figure 5B). As the carbon fibers located only on the sheets’ top and bottom surfaces had electrospun PAN nanofibers, AFM scans were exclusively performed between two faces of these carbon fiber sheets, as depicted in the interphases’ optical microscope images (Figure S5, Supporting Information).

We used the AFM tip to perform a force‐indentation study at every point within a considered region around carbon fiber‐matrix interphase for each sample condition and the representative indentation versus force curves are shown in Figure 5C. This yields local stiffness maps generated from the fitting of a Hertz model^[^ ^61^ ^]^ to each force‐indentation curve having the local Young's modulus as a free‐fitting parameter. Given that the PAN‐ABS polymer chains are covalently bonded at the fiber‐matrix interphase through heat treatment, we hypothesized the formation of distinct interphases around the carbon fiber with intermediate stiffness, schematically shown in Figure 5D. We used the topography images (Figure 5E–G) to generate a binarized mask (Figure 5H–J) ruling out the pixels that were directly on the carbon fiber (too stiff to be characterized in this fashion). The AFM‐derived raw *E* maps of the three different specimens (i.e., neat ABS/ Baseline, (PAN‐ABS)_150 °C_, and (PAN‐ABS)_250 °C_) are shown in Figure S6 (Supporting Information) were processed through a 2D Gaussian smoothing kernel with a standard deviation of 0.5 and displayed in Figure 5K–M. The local Young's modulus maps (*E*) of the neat ABS‐carbon fiber composite (Figure 5K) identify a single stiffness region corresponding to the matrix, delineated in blue (E ≈0.85 GPa). (PAN‐ABS)_150 °C_ composite (Figure 5I) has similar features with a single stiffness region. A small mechanical stiffening can be observed in some areas, likely due to surface debris affecting AFM measurements. However, a clear interphase region was not detected. In contrast, a dramatic change in the stiffness map was observed in the (PAN‐ABS)_220 °C_ specimen (Figure 5M), where a localized interphase region (yellowish‐red) with higher *E* (*E* >15 GPa) was spotted. Only the (PAN‐ABS)_220 °C_ specimen exhibits such a property because its carbon fiber‐matrix interphase contains a covalently bonded PAN‐ABS polymer system, which has a superior *E* than neat ABS and (PAN‐ABS)_150°C_ as previously found in Figure 4F.

In the continued analysis of Figure 5M, additional image processing techniques were employed, specifically by normalizing all image pixels within the interphase region to a scale ranging from 0 to 1. Here, a value of 0 represents a softer region, while 1 signifies the stiffest area. This processing revealed a distinct interphase surrounding the carbon fiber region with a stiffness gradient, starting at 1 (brown‐red) and gradually decreasing to 0.1 (blue). Moreover, a transition stiffness region was identified with a greenish‐yellow appearance that extends between the interphase and the matrix, indicative of a stiffness value of ≈0.5. The thickness of these regions falls within the range of 0.5–4 µm, respectively, as depicted in Figure S7 (Supporting Information). Importantly, the measured thickness of the brown‐red region closely corresponds to the deposition thickness of PAN nanofibers shown in Figure S4D (Supporting Information). Nonetheless, it is imperative to recognize the inherent qualitative characteristics of these measurements, as they may be influenced by a multitude of factors, including the non‐uniform deposition of PAN nanofibers onto carbon fiber surfaces, the potential displacement of nanofibers during the dip‐coating process in the ABS‐acetone emulsion, among other variables. Several factors may influence the size of these interphases, including the deposition thickness of PAN nanofibers, the duration of deposition, electrospinning voltage, nanofiber diameter and surface area, and the temperature and duration of heat treatment, among others. Despite these uncertainties, the AFM‐based nanoindentation study indicates the formation of an intermediate stiffness region surrounding the carbon fiber surfaces. A similar conclusion was reached about the PAN‐ABS covalent bonding during solid‐state NMR (Figure 4L), solubility study (Figure 4K), and rheological characterization (Figure 4A–D). It should be noted that the quantitative comparison of the AFM‐derived *E* values of the specimens with the one obtained via tensile tests (Figure 4F) should be made with care as it is a well‐known issue in nanomechanical characterization, where the quantitative discrepancies of the results arise from the variability of experimental parameters (e.g., AFM tip radius ^[^ ^62^ ^]^). However, the relevant information relies on the relative change in interphase stiffness with heat treatment temperature‐driven interphase formation as found via an AFM‐based indentation study.

Overall, the AFM results demonstrate that hierarchical PAN fibers, when covalently bonded with ABS matrix at carbon fiber‐ABS matrix interphase through proper heat treatment, create a region with intermediate stiffness that could provide a better fiber‐to‐matrix load transferring pathway.

### 2.3 MD Simulation

MD simulation explores the molecular mechanics of how covalently bonded PAN‐ABS molecules at the fiber‐matrix interphase could influence the fiber‐matrix interfacial strength. To reduce the computational demand, a planar graphene sheet with 20,125 carbon atoms of size 12 × 12 nm^2^ was modeled as a substitute for carbon fiber. At the molecular level, the carbon fiber diameter (≈5 µm) is significantly larger than the individual polymer chains, leading to the consideration of a planar graphene sheet as a more suitable representation for carbon fibers. In this context, the analysis does not take into account the presence of any impurities and defects that may be present in carbon fibers/ graphene sheets. 211 ABS and 3 PAN molecules with smaller chain lengths were considered. These numbers were used based on the molecular weights of the considered PAN and ABS chains, aiming to closely mimic the composition of our experimental specimens, where the final composite comprises 70 wt.% carbon fibers, with 0.01 wt.% PAN, while the remaining content consists of ABS. Four cases were modeled, where the baseline has only ABS molecules, and Cases 1–3, where the number of covalently bonded PAN‐ABS molecules gradually increased according to the PAN‐ABS covalently bonded molecule template shown in Figure S8 (Supporting Information). The heterocycle formed through PAN‐ABS covalent bonding via heat treatment (Figure 3B) was excluded from consideration, as its presence could potentially influence the interfacial strength. However, the primary focus of this computational study was to investigate the variation of interfacial energy specifically attributed to PAN‐ABS covalent bonding. The experimental section and Supplementary Materials show the modeling details and the theoretical formulation.

**Figure 6:**
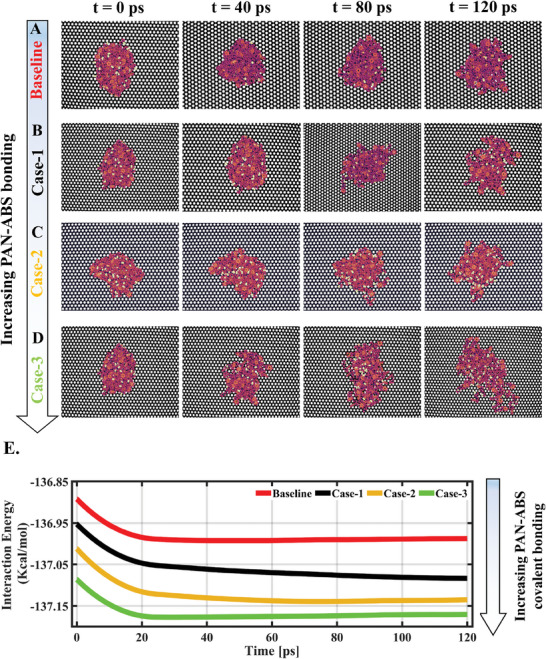
MD simulation captures the relaxation of polymer chains with different numbers of covalently bonded PAN‐ABS molecules on the graphene surface. MD simulation snapshots of the interaction between polymers and graphene for A) baseline, B) Case‐1, C) Case‐2, and D) Case‐3. E) The evolution of interaction energy between each modeled polymer case and the graphene sheet during the relaxation obtained from 120 ps of MD simulation.

**Figure** **6**A–D represents snapshots of the graphene sheet and polymer systems with various covalent bonding numbers at their initial and the steady‐state confirmation process obtained from MD simulation. In all instances, our observations indicated that the polymer chains exhibited a stretching and migrating behavior toward the graphene sheet, eventually dispersing, and spreading across its surface. In the baseline case, the polymer chains did not exhibit significant unentanglement or dispersion on the graphene sheet. For the baseline case, the effective radius of the polymer system at t = 0 ps was ≈4 nm, and it only marginally increased to ≈4.8 nm (≈20%). In Case‐1, Case‐2, and Case‐3, the effective radius increased by ≈32%, 36%, and 50%, respectively, compared to their respective initial effective diameters. These results validate that covalently bonded PAN‐ABS molecules have a better affinity to graphene sheet which could be a key factor for improving the interfacial strength of the FRPCs.

For a more quantitative conclusion, the dynamic behavior of the graphene sheet‐polymer system was evaluated by calculating the interaction energy (*E*_interaction_) using Equation (1):

(1) $$E_{\mathrm{interaction}}=E_{\mathrm{total}}-E_{\mathrm{graphene}\mathrm{sheet}}-E_{\mathrm{polymer}}$$

where *E*_total_, *E*_graphene sheet_, and *E*_polymer_ are the potential energies of the total system, only graphene sheet, and the polymer system, respectively. Figure 6E shows the evolution of *E*_interaction_ due to relaxation at 25 °C. In all cases, *E*_interaction_ steeply goes down in the initial phase of the simulation. After ≈20 ps, although a slow yet gradual reduction in *E*_interaction_ was observed due to polymer relaxation, no significant depletion was noticed beyond 90 ps signifying the systems’ equilibration. It is also clear that Case‐1 has a lower *E*_interaction_ (−137.083 Kcal mol^−1^) than the baseline (−136.988 Kcal mol^−1^) after equilibration. In addition, with the increase in covalently bonded PAN‐ABS molecule numbers (e.g., Case‐3), the *E*_interaction_ further drops to −137.17 Kcal mol^−1^. Although small, such a consistent downtrend in *E*_interaction_ with PAN‐ABS covalent bonding clearly signifies that more PAN‐ABS bonding would be favorable to offer better interfacial strength to the bulk composites. This model establishes a guideline for interphase design via the proposed hierarchical nanofiber‐polymer matrix covalent bonding scheme.

The model will be further improved in the future to serve as a digital guideline for interphase designing via the proposed hierarchical nanofiber‐polymer matrix covalent bonding scheme.

### 2.4 In‐Plane Shear Strength Study

**Figure 7:**
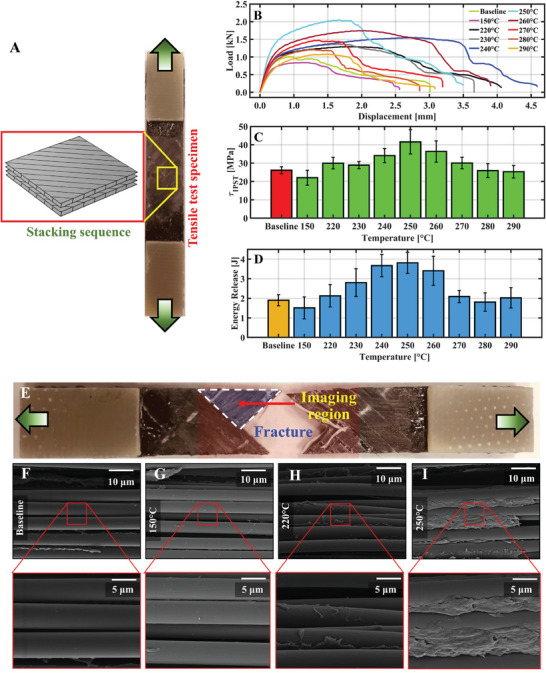
In‐plane shear strength test was performed to characterize the composites’ fiber‐matrix interfacial properties. A) Carbon fiber‐ABS composites with [+45°/‐45°]_2_ were subjected to monotonic tensile strain for in‐plane shear strength characterization. B) Their typical displacement vs. strain behavior is showcased. C) and D) summarize their τ_IPST_ and fracture energy release, respectively. E) The fractured surface of the composites was examined under SEM. The fractured surfaces’ morphology for F) neat ABS, G) (PAN‐ABS)_150 °C_, H) (PAN‐ABS)_220 °C_, and I) (PAN‐ABS)_250 °C_ are shown.

Our last experiment demonstrates the competence of PAN fibers’ covalent bonding with the polymer matrix at the carbon fiber‐ABS matrix interphase to enhance the toughness of the bulk composites. Previously, we found through the rheological study (Figure 4A–D) and solubility test (Figure 4K) that a higher heat treatment temperature could assist more PAN‐ABS bonding. Furthermore, the MD result (Figure 6E) suggested that more PAN‐ABS covalent bonding could enhance interfacial strength. Therefore, additional improvement in bulk composites’ mechanical performances could be achieved by optimally selecting the heat treatment temperature. Composite laminates with symmetric unidirectional carbon fiber sheet laid up at [+45°/−45°]_2_ were prepared through different heat treatment temperatures and their in‐plane shear strength (τ_*IPST*_)^[^ ^63^ ^]^ was characterized as a direct measure of carbon fiber‐matrix interfacial strength (**Figure** **7**A).

Figure 7B shows the representative load‐displacement response for all the tested specimens. All these graphs exhibit a linear elastic deformation region, followed by yielding before failure. We observed that the peak load and the yielding of the composites increase with the heat treatment temperature. The energy release prior to the complete failure of the composites was evaluated by calculating the area under the load‐displacement curves. The average τ_IPST_ and energy release results are summarized in Figure 7C,D, respectively. We found that both these parameters exhibit a similar trend with heat treatment temperature. For example, (PAN‐ABS)_220 °C_ composites have ≈15% and ≈12% increment in τ_IPST_ and energy release than neat ABS composites that gradually increased to a maximum of ≈60% and ≈100%, respectively, as found in (PAN‐ABS)_250 °C_. Such improvement is justified as the number of covalently bonded PAN‐ABS molecules at the fiber‐matrix interphase increases with heat treatment temperature, which has better adhesion with the carbon fibers.

To experimentally validate that, we examined the fractured surfaces (Figure 7E) of the tested specimens under SEM. Micrograms at different magnifications of four different composites are shown in Figure 7F–I. Although complete removal of the polymer matrix from the fiber surface was observed in neat ABS and (PAN‐ABS)_150 °C_ composites, (PAN‐ABS)_220 °C_ composites always had a rough fracture plane with some polymers adhered to the fiber surfaces. The adhered polymer quantity was more prevalent in (PAN‐ABS)_250 °C_ composites. These results make sense as the (PAN‐ABS)_250°C_ specimens have more covalently bonded PAN‐ABS molecules than (PAN‐ABS)_220 °C_ offering an enhanced polymer‐carbon fiber adhesion as suggested by our MD simulation (Figure 6D).

The decrement in τ_IPST_ and fracture energy beyond 250 °C was due to the thermal degradation of ABS as found during its thermogravimetric analysis (Figure S9, Supporting Information). Nonetheless, these results prove that covalent bonding of hierarchical PAN fibers with the ABS matrix at the carbon fiber‐matrix interphase carried out at an optimally selected heat treatment temperature significantly enhances the interfacial strength and toughness of the bulk composites.

## 3 Conclusion

To date, various fiber‐matrix interphase reinforcing techniques have been proposed; however, no research has been conducted on hierarchical structures’ covalent bonding with the polymer matrix for toughening the interphase. The results presented in this manuscript establish the fundamental understanding of nanofiber scaffolding at the core fiber‐matrix interphase and its covalent bonding with the polymer matrix to result in a composite with outstanding fiber‐matrix interfacial strength and toughness. Our approach is straightforward yet practical to produce a composite with superior fiber‐matrix interfacial properties. We demonstrated our hypothesis on a composite that has PAN and ABS as hierarchical structure and polymer matrix, respectively. These constituents were utilized as they have compatible nitrile groups to facilitate PAN‐ABS covalent bonding upon suitable heat treatment. We deployed electrospinning as a scalable, low‐cost method to fabricate hierarchical PAN nanofibers and integrate them on different substrates. SEM imaging revealed that electrospinning parameters used in this study were able to produce a randomly distributed network of PAN nanofibers with consistent morphology. PAN and ABS were shear‐mixed and hot‐pressed at various temperatures resulting in PAN‐ABS composites. We verified PAN‐ABS covalent bonding through rheology, mechanical, and solubility tests. PAN‐ABS composites’ *η* and *G*’ are orders of magnitude higher than neat ABS as found through rheology study. The uniaxial tensile test reported that hierarchical PAN fibers enhanced the ABS's *E*, ultimate strength, and fracture toughness by ≈60%, ≈20%, and ≈50%, respectively. We found that the hierarchical architecture of the PAN fibers plays a crucial role in achieving such improvement in mechanical properties. The solubility test pointed out that PAN‐ABS composites heat treated at a suitable temperature did not dissolve in a solvent, while neat PAN, ABS, and uncrosslinked PAN‐ABS composites were dissociated. We also noticed that the gel fraction of the PAN‐ABS composites increases with the heat treatment temperature. Besides these indirect methods, FTIR and solid‐state NMR spectroscopy results support the hypothesis of interphase rigidization for melt‐mixed composites at temperatures which facilitate cyclization and potential covalent PAN‐ABS interaction. While all these results are crucial for understanding the covalently bonded PAN‐ABS composites’ behavior, it is worth noting that these dramatic improvements were achieved by introducing very little PAN nanofibers (as low as 0.01 wt.%) in the ABS and processing them together within the ABS's processing temperature. This work will pave a new avenue toward 3D‐printed ABS structures with higher mechanical properties.

When integrated at the fiber‐matrix interphase of an FRPC system, these nanofibers, covalently bonded with ABS, create an interphase with intermediate stiffness, as found through AFM indentation study. Such interphases serve as a co‐continuous network at discontinuous‐type fiber‐matrix interphases, offering a better fiber‐matrix load‐transferring pathway. We performed MD simulation to understand the fiber‐matrix interaction behavior at the molecular level. The computational findings unveiled that covalently bonded PAN‐ABS molecules have a higher affinity to adhere to the fiber surface. This platform could be useful to determine the required covalent bonding level and the associated heat treatment temperature to achieve an FRPC with desired interfacial properties. These FRPCs with nanoengineered fiber‐matrix interphases exhibit superior in‐plane shear strength and toughness in FRPC (≈60% and 100% improvement, respectively). The key criterion for these profound mechanical improvements is the ability of the nitrile group within the resin to undergo the necessary reactions and form covalent bonds with the nitrile groups in the PAN nanofibers. It is envisaged that this methodology can be applied to other nitrile‐containing polymer matrices, such as styrene‐acrylonitrile copolymer thermoplastic and acrylonitrile‐butadiene copolymer rubber, possibly having widespread applicability in traditional FRPCs. Our technique has the potential to surpass the chemically intensive processes used these days for strengthening the core fiber‐matrix interphases. We envision that the findings reported in this paper will open up a new horizon of fiber‐matrix interphase design to achieve high‐performance composites, especially for aerospace and automotive structures.

## 4 Experimental Section

### Materials

IM2 unidirectional carbon fiber sheets and GP35 ABS pallets used in this study were sourced from Composite Envisions, LLC, and M‐Holland, LLC, respectively. The PAN powder contains copolymers of 95.36 mol.% acrylonitrile and 4.64 mol.% methyl acrylate, which had a molecular weight of 126 kDa and polydispersity of 2.23. Acetone, dimethylformamide (DMF), CHCl_3_, and C_2_H_6_OS were purchased from Fisher Scientific LLC.

### Electrospinning

A 5 wt.% PAN dope was prepared by mixing ≈0.99 g of PAN powder in 20 mL of DMF for electrospinning. The mixture was mechanically stirred with a magnetic stirrer at 300 rpm for 48 h until all the PAN powder was dissolved, resulting in a clear golden‐yellowish solution that was utilized for electrospinning. The dope was loaded into a syringe with a blunt tip needle for the electrospinning process. The PAN nanofibers were collected on two different substrates (i.e., aluminum foil, unidirectional carbon fiber sheets) and used in various experiments.

Unidirectional carbon fiber sheet/ aluminum foil (457 × 152 mm^2)^ was wrapped around a cylindrical collector that was electrically grounded and rotated at ≈115 rpm. A low rpm provided random and even deposition of PAN fibers on the carbon fiber sheets. A 6.5 kV DC voltage was used for electrospinning and the distance between the mold and the needle tip was set to ≈14 cm. The electrospinning process was performed for ≈30 s with a syringe pump rate of 0.75 mL h^−1^. The fabrication setup is schematically presented in Figure 2A.

For morphological characterization through SEM imaging, A 10‐nm‐thick gold particle layer was deposited on PAN‐coated substrates. A TESCAN MIRA3 scanning electron microscope operated at 10 kV was used for SEM imaging.

### Specimen Preparation

PAN‐ABS composites with ≈0.01 wt.% PAN fibers were prepared by shear‐mixing electrospun PAN nanofibers with ABS pellets (Figure 3C) at 150 °C. The total shear‐mixing time was limited to 30 min with a mixing speed of 60 rpm. ABS pellets were added into the mixer and shear mixing was performed for 12 min followed by addition PAN fibers and their shear mixing. PAN fibers deposited on aluminum foil was used in this step. The shear‐mixed PAN‐ABS beads were hot‐pressed (≈958 kPa, 30 min) at different temperatures (Figure 3D). The hot‐pressed PAN‐ABS plates (Figure 3E) were cut into small pieces and used in solubility tests. These small pieces were pulverized into powder using a cryogenic grinder for FTIR and solid‐state NMR. PAN specimens used for NMR spectroscopy was directly sampled from PAN powder. From a (PAN‐ABS)_150 °C_ plate, 10‐mm‐diameter, 1‐mm‐thick circular discs were cut out for the rheology test. Type V dogbones^[^ ^56^ ^]^ were cut from the hot‐pressed PAN‐ABS plate using a ProtoMax water jet cutter for mechanical tests.

Composite laminates were fabricated by stacking unidirectional carbon fiber sheets in different sequences with both faces coated with electrospun PAN fibers. AFM specimens had two layers of fiber stacked in the same direction (Figure 5B), whereas in‐plane shear strength test specimens had four layers of unidirectional carbon fiber sheets symmetrically stacked at ± 45° (Figure 7A). The sheets were dip‐coated in 10 wt.% ABS emulsion in acetone (Figure 5A) and dried at room temperature for ≈24 h. The ABS‐acetone emulsion was prepared by dissolving ≈52.3 g of ABS pellets in 600 mL of acetone using a magnetic stirrer (≈500 rpm). The mixing was performed at room temperature for≈24 h. ABS‐coated carbon fiber sheets were hot‐pressed under different temperatures (150°C, and 220°C to 290°C at 10°C intervals) and constant pressure (≈958 kPa) for ≈30 min. Composites with two layers of carbon fiber sheets were cut into ≈10 × 10 mm^2^ strips and cured with epoxy resin for AFM scanning. These epoxy pucks were polished to obtain a smooth surface for AFM scanning. The polishing steps were crucial to preparing the sample for AFM‐based nanoindentation study. More detail can be found in Supporting Information. Three specimens were prepared for AFM study (i.e., neat ABS, (PAN‐ABS)_150 °C_, (PAN‐ABS)_220 °C_). On the other hand, the four‐layer carbon fiber‐contained composite panels were cut into 100 × 10 mm^2^ rectangular pieces with the water jet cutter and tabbed with 25 × 10 mm^2^ garolite strips for in‐plane shear strength test (Figure 7A).

### Testing Protocols

All rheological measurements were performed using a DHR‐3 rotational rheometer (TA Instruments, New Castle, DE) equipped with 8‐mm‐diameter stainless steel parallel plates under an air atmosphere. The rheometer was commanded to maintain a 1 N compressive force to ensure zero slip of the composites during testing. The temperature was gradually raised (10°C min^−1^) to the desired level, followed by a ≈30 min isotherm to facilitate the PAN‐ABS in situ chemical coupling. After that, the rheometer was commanded to cool down to 150 °C and waited for ≈1 min before starting the test. Thereafter, the oscillatory frequency sweep was performed with the shear strain of 0.1% to observe the dynamic response of a PAN‐ABS composite in the angular frequency range from 0.01 to 100 rad s^−1^. The shear strain magnitude was set from the linear viscoelastic response regime obtained from the amplitude–frequency sweep study. Neat ABS and PAN‐ABS composites heat treated at four different temperatures (220–250 °C in 10 °C intervals) were subjected to the rheological test.

The gel content experiment was performed by solvent extraction method.^[^ ^58^ ^]^ Neat ABS, neat PAN, (PAN‐ABS) composites heat‐treated at 150, 220, 230, 240, and 250 °C were subjected to gel content experiment. Each sample was pre‐weighed (*M*_i_) prior to extracting them in a 15 mL solvent mixture of DMSO/CHCl_3_ (1:1, w/w) for 48 h in a 20 mL glass vial at 60 °C. Thereafter, the specimens were dried in a vacuum oven at 120 °C for 24 h and weighed (*M*_d_). Gel fraction was calculated using Equation (2).

(2) $$\mathrm{Gel}\mathrm{fraction}=\frac{M_{d}}{M_{i}}\times 100$$

All mechanical tests were performed in a displacement‐controlled format using an MTS Alliance RT/5 tensile load frame instrumented with a 5 kN load cell and tested in tension until their failure using a displacement rate of 1 mm min^−1^ until composite failure and load versus crosshead displacement data were sampled at 10 Hz. The average width and thicknesses of the five specimens from each set were measured and used to evaluate the composites’ tensile and in‐plane shear strength. A total of six specimens were examined within each set.

For the solid‐state NMR, ^1^H T_1_ inversion recovery measurements were performed with a Bruker Avance III spectrometer operating at 400 MHz ^1^H frequency under 10 kHz MAS in a 3.2 mm triple resonance probe. A “bulk” T_1_ was considered given the lack of spectral resolution for the sample by integrating the entire spectrum for T_1_ analysis. ^13^C NMR experiments were performed using 700 MHz VNMRS spectrometer systems equipped with a T3 Varian probe. The data were collected using a spin echo sequence under 22.5 kHz MAS, a recycle delay of 75 s, and ≈2,000 scans.

The AFM force curve mapping shown in Figure 5 was performed using an MFP3D from Asylum research Oxford Instruments in air. Using the TESPA‐V2 tips from Bruker with a measured spring constant of 29.07 N m^−1^ using the thermal noise method.

In the in‐plane shear strength test, τ_IPST_ was calculated using Equation (3).

(3) $$\tau_{IPST}=\frac{P_{\max}}{2A}$$

where *P*_max_ is the maximum load obtained from the load‐displacement curve and *A* is the cross‐sectional area of the specimen.

### MD Simulation

ABS and PAN chains shown in Figure 3A were considered in the MD simulation. *x*, *y*, and *z* for one ABS molecule were equal to one. The number of repeating units (*n*) for PAN and ABS was assumed to be equal to 10 and 1, respectively. The PAN‐ABS covalent bonding was manually performed according to the reaction template, shown in Figure S8. In Case‐1, 10 ABS chains were attached to one PAN chain. The number of such covalently bonded PAN‐ABS molecules was increased to two and three in Case‐2 and Case‐3, respectively. The size of the graphene sheet was selected as 12 × 12 nm^2^ consisting of 20125 carbon atoms.

The polymer system and the graphene sheet were first equilibrated separately before placing the polymer near the graphene sheet, maintaining a minimum distance of 2 nm. This was followed by the minimization of the entire system to remove any distortions of atomic arrangement during the construction and placement of the molecules. The optimized system was then equilibrated by raising the temperature from absolute zero to 150 **°**C for 50 ps followed by an equilibration for another 50 ps. The system was then cooled to 25 °C within 50 ps and equilibrated for 120 ps. The entire simulation was carried out under the NVT ensemble utilizing the Nose Hoover thermostat, and the periodic boundary condition was utilized in all directions.^[^ ^64^ ^]^ During the entire equilibration process, the graphene sheet was made rigid, avoiding any thermal wrinkles or distortion and was fixed to a point to prevent any rigid body movement. Similar steps were implemented during the equilibration process of individual polymers as well as for only graphene sheet. The evolution of *E*_interaction_ for the final 120 ps was reported with an origin shifting to t = 150 ps. The systems’ energy was extracted at every 0.1 ps. Moving average filtering with a sliding window length 3000, was utilized to process the MD simulation‐generated *E*_interaction_.

## Conflict of Interest

The authors declare no conflict of interest.

## Supporting information

Supporting Information

## Data Availability

The data that support the findings of this study are available from the corresponding author upon reasonable request.

## References

[1] P. K. Mallick , Fiber‐reinforced composites: materials, manufacturing, and design, CRC Press, Boca Raton Florida 2007.

[2] R. Selzer , K. Friedrich , Composites, Part A 1997, 28, 595.

[3] A. T. DiBenedetto , Mater. Sci. Eng., A 2001, 302, 74.

[4] J. K. Kim , Y. W. Mai , Engineered interfaces in fiber reinforced composites, Elsevier, Oxford, U.K 1998.

[5] U. G. K. Wegst , M. F. Ashby , Philos. Mag. 2004, 84, 2167.

[6] Z. Dai , F. Shi , B. Zhang , M. Li , Z. Zhang , Appl. Surf. Sci. 2011, 257, 6980.

[7] M. A. Downey , L. T. Drzal , Composites, Part A 2016, 90, 687.

[8] M. Tanoglu , S. Ziaee , S. H. Mcknight , G. R. Palmese , J. W. Gillespie, Jr. , J. Mater. Sci. 2001, 36, 3041.

[9] P. Zinck , J. Gerard , Compos. Sci. Technol. 2008, 68, 2028.

[10] Y. Dong , Y. Zhu , Y. Zhao , F. Liu , E. Wang , Y. Fu , Composites, Part A 2017, 102, 357.

[11] W. Liu , S. Zhang , B. Li , F. Yang , W. Jiao , L. Hao , R. Wang , Polym. Compos. 2014, 35, 482.

[12] A. G. Atkins , J. Mater. Sci. 1975, 10, 819.

[13] S. H. Deng , X. D. Zhou , M. Q. Zhu , C. J. Fan , Q. F. Lin , eXPRESS Polym. Lett. 2013, 7, 925.

[14] R. L. Oréfice , A. E. Clark , A. B. Brennan , J. Appl. Polym. Sci. 2006, 99, 1153.

[15] Z. Liu , F. Zhao , F. R. Jones , Compos. Sci. Technol. 2008, 68, 3161.

[16] D. J. Marks , F. R. Jones , Composites, Part A 2002, 33, 1293.

[17] P. Photjanataree , Z. Liu , F. R. Jones , Macromol. Mater. Eng. 2012, 297, 523.

[18] F. Vautard , P. Fioux , L. Vidal , F. Siffer , V. Roucoules , J. Schultz , M. Nardin , B. Defoort , ACS Appl. Mater. Interfaces 2014, 6, 1662. 24359478 10.1021/am4045663

[19] E. Bekyarova , E. T. Thostenson , A. Yu , H. Kim , J. Gao , J. Tang , H. T. Hahn , T.‐W. Chou , M. E. Itkis , R. C. Haddon , Langmuir 2007, 23, 3970. 17326671 10.1021/la062743p

[20] L. Chen , H. Jin , Z. Xu , M. Shan , X. Tian , C. Yang , Z. Wang , B. Cheng , Mater. Chem. Phys. 2014, 145, 186.

[21] C. C. Bowland , N. A. Nguyen , A. K. Naskar , ACS Appl. Mater. Interfaces 2018, 10, 26576. 30003781 10.1021/acsami.8b03401

[22] C. C. Bowland , S. Gupta , S. M. Rankin , A. K. Naskar , in Nondestructive Characterization and Monitoring of Advanced Materials, Aerospace, Civil Infrastructure, and Transportation XV, SPIE, Bellingham, Washington USA 2021.

[23] S. Gupta , A. K. Naskar , C. C. Bowland , Adv. Mater. Technol. 2022, 7, 2101549.

[24] S. Gupta , A. K. Naskar , C. C. Bowland , in Nondestructive Characterization and Monitoring of Advanced Materials, Aerospace, Civil Infrastructure, and Transportation XVI, SPIE, Long Beach, CA 2022, 12047.

[25] S. M. Rankin , M. K. Moody , A. K. Naskar , C. C. Bowland , Compos. Sci. Technol. 2021, 201, 108491.

[26] J. G. Gonzalez , S. Gupta , K. J. Loh , Proc IEEE Inst Electr. Electron Eng 2016, 104, 1547.

[27] S. Gupta , J. G. Gonzalez , K. J. Loh , Struct. Health Monit. 2017, 16, 309.

[28] M. K. Hossain , M. M. R. Chowdhury , M. B. Salam , J. Malone , M. V. Hosur , S. Jeelani , N. W. Bolden , J. Appl. Polym. Sci. 2014, 131, 40709.

[29] D. Pedrazzoli , A. Pegoretti , K. Kalaitzidou , Compos. Sci. Technol. 2014, 98, 15.

[30] M. A. Ashraf , W. Peng , Y. Zare , K. Y. Rhee , Nanoscale Res. Lett. 2018, 13, 214. 30019092 10.1186/s11671-018-2624-0 PMC6049851

[31] J. Chen , D. Zhao , X. Jin , C. Wang , D. Wang , H. Ge , Compos. Sci. Technol. 2014, 97, 41.

[32] S. Zhu , C.‐H. Su , S. L. Lehoczky , I. Muntele , D. Ila , Diamond Relat. Mater. 2003, 12, 1825.

[33] H. Qian , A. Bismarck , E. S. Greenhalgh , G. Kalinka , M. S. P. Shaffer , Chem. Mater. 2008, 20, 1862.

[34] V. P. Veedu , A. Cao , X. Li , K. Ma , C. Soldano , S. Kar , P. M. Ajayan , M. N. Ghasemi‐Nejhad , Nat. Mater. 2006, 5, 457. 16680146 10.1038/nmat1650

[35] G. J. Ehlert , U. Galan , H. A. Sodano , ACS Appl. Mater. Interfaces 2013, 5, 635. 23281964 10.1021/am302060v

[36] G. J. Ehlert , H. A. Sodano , ACS Appl. Mater. Interfaces 2009, 1, 1827. 20355800 10.1021/am900376t

[37] N. Bhardwaj , S. C. Kundu , Biotechnol. Adv. 2010, 28, 325. 20100560 10.1016/j.biotechadv.2010.01.004

[38] S. Ramakrishna , K. Fujihara , W. E. Teo , T. C. Lim , Z. Ma , An introduction to electrospinning and nanofibers, World Scientific, Singapore 2005.

[39] W. E. Teo , S. Ramakrishna , Nanotechnology 2006, 17, R89. 19661572 10.1088/0957-4484/17/14/R01

[40] A. Baji , Y.‐W. Mai , S.‐C. Wong , M. Abtahi , P. Chen , Compos. Sci. Technol. 2010, 70, 703.

[41] C. Ayres , G. L. Bowlin , S. C. Henderson , L. Taylor , J. Shultz , J. Alexander , T. A. Telemeco , D. G. Simpson , Biomaterials 2006, 27, 5524. 16859744 10.1016/j.biomaterials.2006.06.014 PMC2929953

[42] K. B. Wiles , M.Sc Virginia Polytechnic Institute and State University Blacksburg, Virginia 2002.

[43] P. J. Sánchez‐Soto , M. A. Avilés , J. C. Del Rio , J. M. Ginés , J. Pascual , J. L. Pérez‐RodriGuez , J. Anal. Appl. Pyrolysis 2001, 58, 155.

[44] E. Frank , L. M. Steudle , D. Ingildeev , J. M. Spörl , M. R. Buchmeiser , Angew. Chem., Int. Ed. 2014, 53, 5262. 10.1002/anie.201306129 24668878

[45] Z. Bashir , Carbon 1991, 29, 1081.

[46] X. D. Liu , W. Ruland , Macromolecules 1993, 26, 3030.

[47] J. Simitzis , S. Soulis , Polym. Int. 2008, 57, 99.

[48] X. Liu , Y. Makita , Y.‐L. Hong , Y. Nishiyama , T. Miyoshi , Macromolecules 2017, 50, 244.

[49] T.‐H. Ko , J. Appl. Polym. Sci. 1991, 42, 1949.

[50] T.‐H. Ko , H.‐Y. Ting , C.‐H. Lin , J. Appl. Polym. Sci. 1988, 35, 631.

[51] M. S. A. Rahaman , A. F. Ismail , A. Mustafa , Polym. Degrad. Stab. 2007, 92, 1421.

[52] J. Lin , J. Li , J. Wang , Y. Guan , G. Wang , L. Chen , Polym. Eng. Sci. 2019, 59, E144.

[53] Y. Wang , M. Chen , M. Lan , Z. Li , S. Lu , G. Wu , Polymers 2019, 12, 46. 31905639 10.3390/polym12010046 PMC7023587

[54] N. A. Nguyen , S. H. Barnes , C. C. Bowland , K. M. Meek , K. C. Littrell , J. K. Keum , A. K. Naskar , Sci. Adv. 2018, 4, eaat4967. 30555914 10.1126/sciadv.aat4967 PMC6294600

[55] G. H. Olive , S. Olive , Polym. Bull. 1981, 5, 457.

[56] H. H. Winter , F. Chambon , J. Rhe. 1986, 30, 367.

[57] B. Arash , Q. Wang , V. K. Varadan , Sci. Rep. 2014, 4, 6479. 25270167 10.1038/srep06479 PMC4180807

[58] S. Dhers , G. Vantomme , L. Avérous , Green Chem. 2019, 21, 1596.

[59] M. S. Morales , A. A. Ogale , J. Appl. Polym. Sci. 2013, 128, 2081.

[60] J. Li , F. Chen , L. Yang , L. Jiang , Y. Dan , Spectrochim. Acta, Part A 2017, 184, 361. 10.1016/j.saa.2017.04.075 28535488

[61] H.‐J. Butt , B. Cappella , M. Kappl , Surf. Sci. Rep. 2005, 59, 1.

[62] D. Hoffman , M.S.Mt.E.,Michigan Technological University 2010.

[63] ASTM D 3518, (ASTM International, West Conshohocken, PA).

[64] W. Shinoda , M. Shiga , M. Mikami , Phys. Rev. B 2004, 69, 134103.

