# Supplementary material for: Enhancing Composite Toughness Through Hierarchical Interphase Formation
Source: Adv Sci (Weinh). 2023 Dec 25;11(6):2305642. doi: 10.1002/advs.202305642 (PMC10853716; doi:10.1002/advs.202305642)
Supplement: Supplementary file 1 — Supporting Information [file ADVS-11-2305642-s001.pdf]

ADVANCED SCIENCE
Open Access

Supporting Information

for *Adv. Sci.*, DOI 10.1002/advs.202305642

Enhancing Composite Toughness Through Hierarchical Interphase Formation

*Sumit Gupta, Tanvir Sohail, Marti Checa, Sargun S. Rohewal, Michael D. Toomey, Nihal Kanbargi, Joshua T. Damron, Liam Collins, Logan T. Kearney, Amit K. Naskar and Christopher C. Bowland**

Supplementary Materials for

Enhancing composite toughness through hierarchical interphase formation

Sumit Gupta *et al.*

*Corresponding author. Email: bowlandcc@ornl.gov

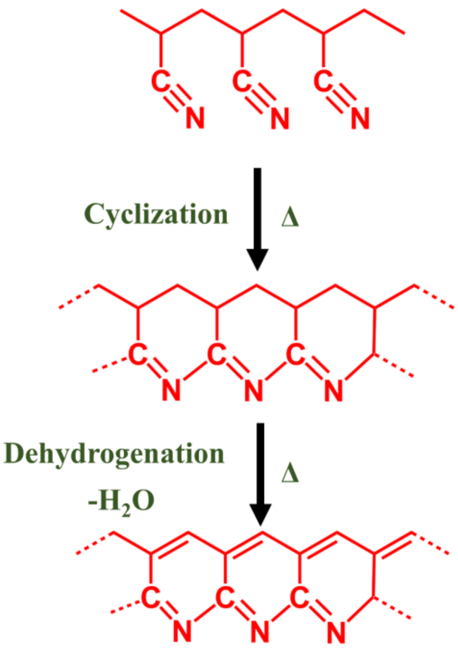

Fig. S1. Oxidative stabilization of PAN.

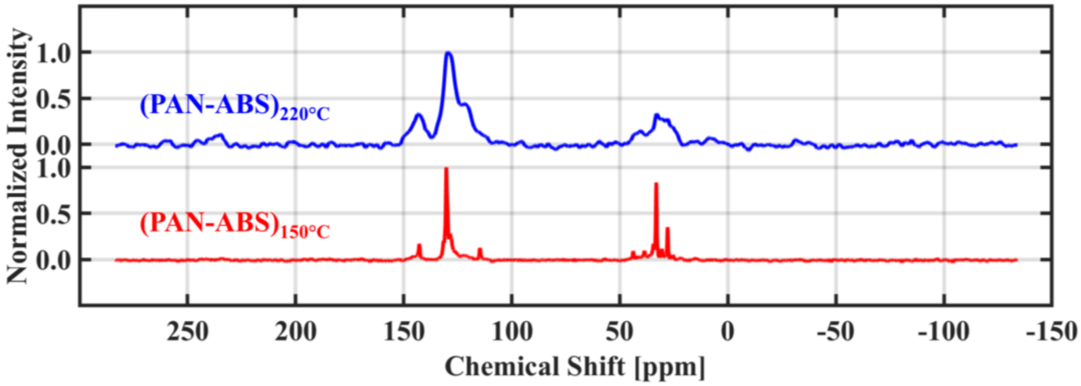

Fig. S2. Solid-state NMR spectra of (PAN-ABS)150°C and $(PAN\text{-}ABS)_{220°C}$ specimens are overlaid.

^{1}H longitudinal relaxation experiments, which are widely used to characterize polymer dynamics [1], were performed on these specimens used in solid-state NMR study and on pristine ABS and PAN. T_1, the relaxation time measured from these experiments, is sensitive to fast motional regimes where motional rates are sensitive to the interaction field frequency (400 MHz here) and $1/T_1$ is maximal when motional rates are resonant with this frequency. T_1 was obtained by plotting the signal intensity as a function of the recovery delay and fitting the data to an appropriate mono-exponential function (equation S1), as shown in fig. S3. The $(PAN\text{-}ABS)_{150°C}$ specimen has a T_1 of ~ 1.5 s, increasing to 6.0 s after heating to 220°C. Neat ABS heated to 220°C has a T_1 of ~ 1.4 s, while neat PAN heated to 220°C exhibits a much longer T_1 of ~ 18 s. This indicates that the two chemical constituents in the $(PAN\text{-}ABS)_{220°C}$ specimens are interacting significantly to produce an intermediate T_1 value indicative of interphase rigidization due to PAN-ABS bonding and cyclization. In general, the relaxation data indicates that the PAN and ABS in $(PAN\text{-}ABS)_{220°C}$ specimen are favorably mixing, exhibiting an intermediate relaxation time between the two samples. The observation supports our hypothesis regarding the occurrence of phase rigidization via covalent bonding and cyclization between PAN and ABS molecules.

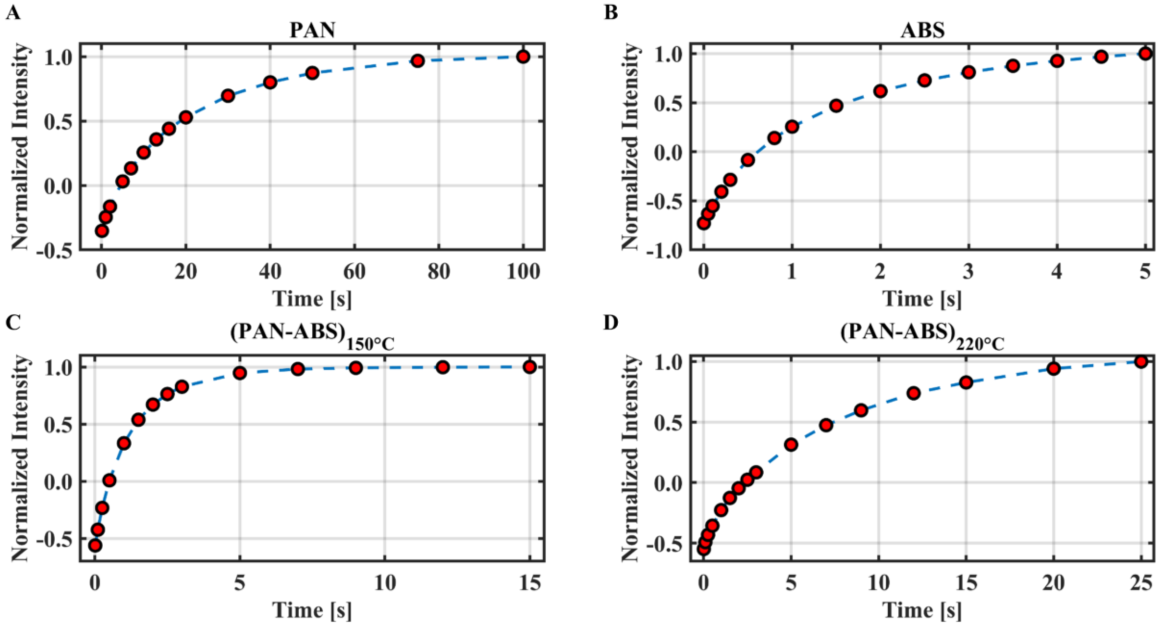

Fig. S3. (A), **(B)**, **(C)**, and **(D)** are the plots obtained by fitting the solid-state NMR data with a mono-exponential function to extract the T_1 for PAN, ABS, $(PAN\text{-}ABS)_{150°C}$ and $(PAN\text{-}ABS)_{220°C}$ specimens, respectively.

$$I_o + P \times \exp(-t/T_1) \qquad (S1)$$

Here, I_o represents the recovery of the NMR signal intensity, P represents the initial magnetization, t is the time delay between the pulse and the acquisition of the signal.

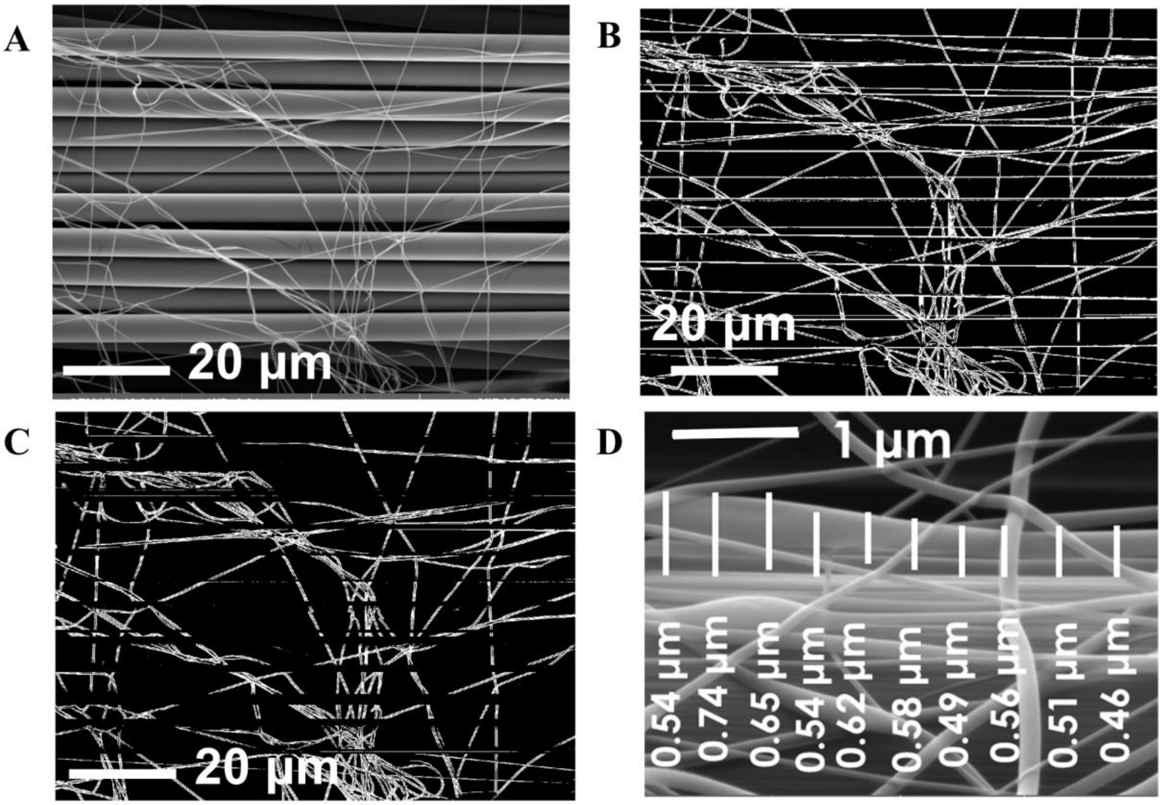

Fig. S4. **(A)** SEM image of the PAN coated carbon fibers, **(B)** the corresponding binary, and filtered image are shown. **(D)** shows the thickness of PAN deposition on carbon fiber.

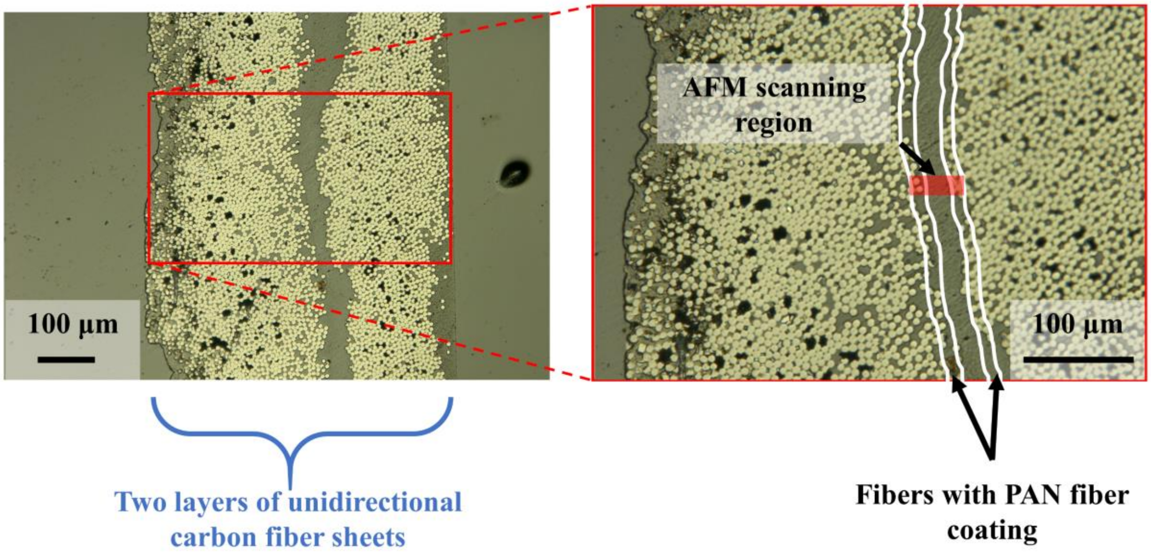

Fig. S5. Optical microscope images at a different magnification of the FRPCs with two layers of unidirectional carbon fiber sheets (cross-sectional view). The targeted AFM scanning region is highlighted with white lines.

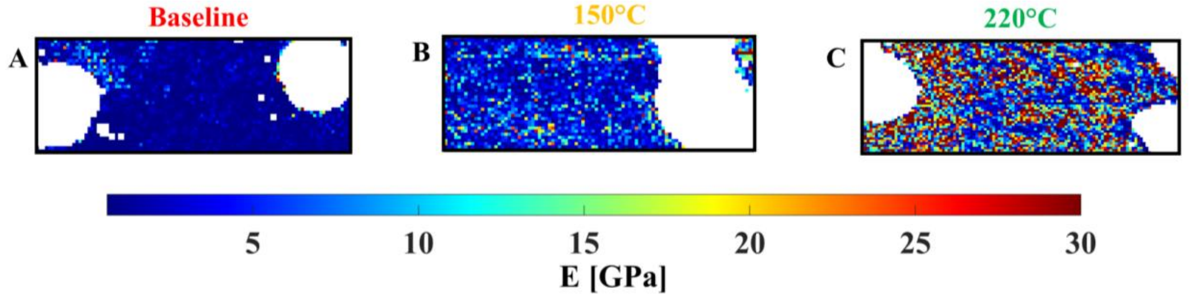

Fig. S6. Raw AFM indentation test maps of the PAN-ABS carbon fiber composites heat-treated at different temperatures.

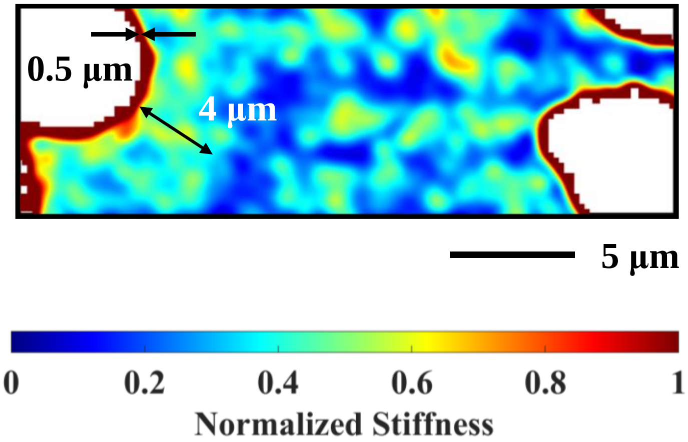

Fig. S7. Interphase thickness measurement via image processing.

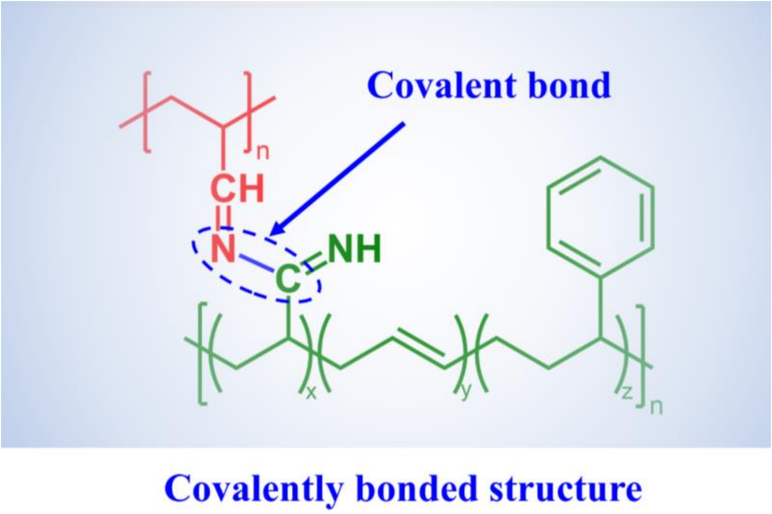

Fig. S8. PAN-ABS covalently bonded structure used as the template in MD simulation.

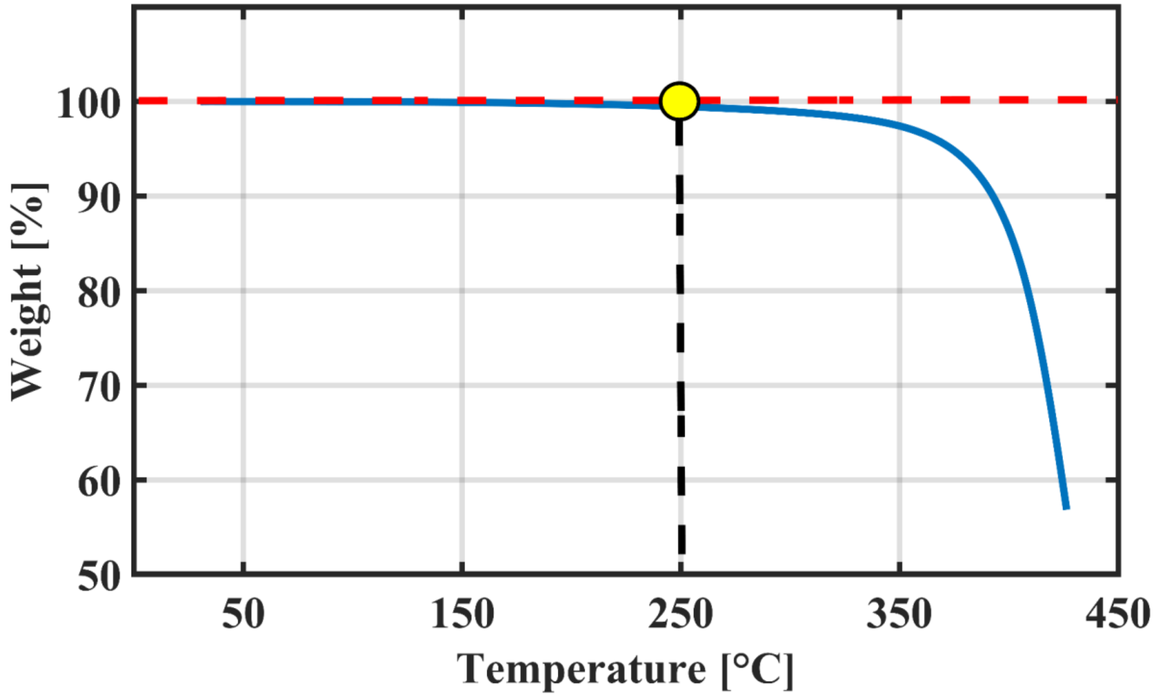

Fig. S9. Thermogravimetric analysis plot of neat ABS.

Herman's Orientation Factor (HOF) Calculation

HOF is a metric that describes the line structure relative to a reference direction that can vary between -0.5 to 1. Here a near zero value will indicate a random orientation of the FFT component. Mathematically, HOF can be defined using equation S2.

$$HOF = \frac{1}{2}\left(3\left\langle \cos^2 \varphi \right\rangle - 1\right) \tag{S2}$$

<> indicate a spatial average to account for the spatial distribution of the orientation degree and can be calculated using equation S3.

$$\left\langle \cos^2 \varphi \right\rangle = \frac{\int_0^{\pi/2} I(\varphi)\cos^2\varphi \sin\varphi d\varphi}{\int_0^{\pi/2} I(\varphi)\sin\varphi \, \mathrm{d}\varphi} \tag{S3}$$

where azimuth angle φ is the angle between the orientation reference axis and the component's direction, and $I(\varphi)$ is the intensity profile of anisotropy as a function of φ from zero to π/2. More detail about the HOF calculation can be found in [2-3].

Morphological Characterization via Image Processing

For a more quantitative assessment of the distribution of PAN fibers, the area of carbon fiber surfaces covered by the PAN fibers by electrospinning (fig. S4A) was estimated through image processing. The image processing algorithm works as follows: first, the scanning electron microscope (SEM) images were converted into binary images where the background and the PAN fibers were marked as black (pixel value = 0) and white (pixel value = 1), respectively (fig. S4B). Second**,** from the binary image, only the pixels corresponding to the PAN fibers were selected. Artifacts from the carbon fibers were excluded from the binary image by selecting only the image pixels with different abscissa assuming that the carbon fibers are horizontally aligned (fig. S4C**)**. The number of pixels corresponding to the PAN fibers was normalized with the total number of image pixels in the image to obtain an area ratio. The image processing result revealed that ~ 10.6% of the carbon fiber surface was covered by PAN fibers during electrospinning, thereby validating the ultra-low concentration of PAN fibers within the bulk composites. This result says that composites (60 × 10 mm^2) with single- and double-layer PAN fibers would have a total of ~ 63.6 mm^2 and ~ 127.2 mm^2 PAN-covered surfaces, respectively.

In order to determine the actual *wt*.% of the PAN in the final composites, the deposition thickness was estimated through another customized image processing software. In this case, the SEM image shown in fig. S4D, was acquired by tilting the SEM stage by ~ 90˚. The deposition thicknesses were measured at ten equidistantly spaced locations along the fiber axis (fig. S3D). The average deposition thickness was ~ 0.557 μm. When multiplied by the previously computed PAN fiber areas, the estimated average thickness predicts the total volume of the PAN fibers within the composites, which, if multiplied by the PAN fibers' density (1.18 g/cm^3), approximates the PAN fiber mass within the final composites. The extracted PAN fiber mass was normalized with the total weight of the composites to obtain the *wt*.% of PAN fibers within the bulk composites. According to our analysis, our PAN-enhanced composites have ~ 0.026 *wt*.% of PAN fibers.

Polishing Procedures for Sample Preparation in AFM-based Nanoindentation Study

Silicon carbide polishing surfaces (particle sizes of 400, 600, and 800 µm, respectively) were utilized in three consecutive steps (ran for 1, 2, and 1 minute, respectively) using an automatic polisher with a controlled load on the specimens. A constant axial load of 18 N was applied to the rotating specimen holder while the base plate rotated in the same direction at a speed of 300 and 60 rpm, respectively. During polishing, water was employed as a coolant. Subsequently, diamond paste with a particle size of 3 µm and an alumina solution with a particle size of 0.05 µm were employed as abrasives with respective axial load of 22 N and 14 N. For these final polishing steps, each lasting 1 minute, the base plate maintained a constant speed of 150 rpm, while the specimen holder rotated at 60 rpm. The plates rotated in the same direction when using diamond paste and in opposing directions when using alumina suspension for polishing.

Molecular Dynamics (MD) Modeling Background

In order to start with the MD process, there is a need to select an appropriate potential to perform the simulations and to calculate the potential energies. Previous literature showed that non-reactive potentials yielded better results when mimicking the non-bonded interaction between planar graphene sheet and polymer. Therefore, instead of using reactive potential *ReaxFF* to study the interaction between polymers and graphene sheet [3], we used the non-reactive PCFF potential [5]. Being a non-reactive potential, *PCFF* does not allow the formation of the covalent bond between the graphene sheet surface and the polymer atoms. Hence, the interaction was limited to only non-bonded interactions studied following the systematic variation of representative crosslinked nitrile groups as schematically shown in fig. S8. It should be noted that the covalently bonded structure shown in Fig. 1 was not considered in MD simulation as we wanted to explore the effect of PAN-ABS intermolecular covalent bonding effect on the graphene sheet-polymer interaction energy. Like all other class 2 potentials, the total potential energy in a system is the summation of various terms, as shown in the following equations below.

$$E_{total} = E_{bond} + E_{angle} + E_{dihedrals} + E_{vdWaals} + E_{coulomb} \quad \text{(S4)}$$

E_{angle} is given by equation S5.

$$E_{angle} = E_a + E_{bb} + E_{ba} \quad \text{(S5)}$$

E_a, E_{bb}, *and* E_{ba} are defined in equations S6-S8, respectively.

$$E_a = H_2(\theta - \theta_0)^2 + H_3(\theta - \theta_0)^3 + H_4(\theta - \theta_0)^4 \quad \text{(S6)}$$

$$E_{bb} = M_2(r_{ij} - r_1)(r_{ij} - r_2) \quad \text{(S7)}$$

$$E_{ba} = N_1(r_{ij} - r_1)(\theta - \theta_0) + N_2(r_{ij} - r_2)(\theta - \theta_0) \quad \text{(S8)}$$

PCFF dihedral energy ($E_{dihedral}$) term is:

$$E_{dihedral} = E_d + E_{mbt} + E_{ebt} + E_{at} + E_{aat} + E_{bb13} \quad \text{(S9)}$$

$$E_d = \sum_{n=1}^{3} K_n (1 - \cos(n\emptyset - \emptyset_n)) \quad \text{(S10)}$$

$$E_{mbt} = (r_{jk} - r_2)(A_1 \cos(\emptyset) + A_2 \cos(2\emptyset) + A_3 \cos(3\emptyset)) \quad \text{(S11)}$$

$$E_{ebt} == (B_1 \cos(\emptyset) + B_2 \cos(2\emptyset) + B_3 \cos(3\emptyset) + (r_{kl} - r_3)(C_1 \cos(\emptyset) + C_2 \cos(2\emptyset) + C_3 \cos(3\emptyset)) \quad \text{(S12)}$$

$$E_{at} = (r_{ij} - r_1)(\theta_{ijk} - \theta_1)(D_1 \cos(\emptyset)(D_1 \cos(\emptyset) + D_2 \cos(2\emptyset) + D_3 \cos(3\emptyset) E_1 \cos(\emptyset) + E_2 \cos(2\emptyset) + E_3 \cos(3\emptyset) \quad \text{(S13)}$$

$$E_{aat} = M(\theta_{ijk} - \theta_1)(\theta_{jkl} - \theta_2) \cos(\emptyset) \quad \text{(S14)}$$

$$E_{bb13} = N(r_{ij} - r_1)(r_{kl} - r_3) \quad \text{(S15)}$$

The van der Waals interaction uses the standard *LJ* potential given by equation S16.

$$E_{vdW} = \varepsilon\left[2\left(\frac{\sigma}{r}\right)^{9} - 3\left(\frac{\sigma}{r}\right)^{6}\right], \text{ r} < r_{cutoff} \quad \text{(S16)}$$

Finally, the coulombic interaction is given by equation S17.

$$E_{Coul} = \sum_{i>j} \frac{q_i}{\varepsilon}\frac{q_j}{r_{ij}} \quad \text{(S17)}$$

where q is the atomic charge, ε is the dielectric constant, and r_{ij} is the i-j atomic separation distance, b and $b^{'}$ are the lengths of two adjacent bonds, θ is the two-bond angle, $\emptyset$ is the dihedral torsion angle, and χ is the out-of-plane angle. b_0, k_i (i = 2, 3, 4), θ_0, H_i (i = 2, 3, 4), $\emptyset^0$, V_i (i = 1, 2, 3). All the parameters are derived according to [5]. The remaining variables used in equations S4-S17 are described in Table S1.

Table S1. Descriptions of variables used in MD modeling

Energy terms	Description
E_{total}	Total Energy
E_{bond}	Energy due to covalent bonds
E_{angle}	Energy due to angles between the atoms
$E_{dihedrals}$	Energy due to dihedrals between the atoms
$E_{vdWaals}$	Energy due to van der Waals interaction
$E_{coulomb}$	Energy due to coulombic interaction
E_{a}	Energy due to only angles
E_{ba}	Energy due to only bond angle interaction
E_{bb}	Energy due to only bond-bond interaction
E_{d}	Energy due to only dihedral interaction
E_{mbt}	Energy due to only middle bond torsion interaction
E_{ebt}	Energy due to only end bond torsion interaction
E_{at}	Energy due to only angle torsion interaction
E_{aat}	Energy due to only angle-angle torsion interaction
E_{bb13}	Energy due to only BondBond13 interaction
q	Atomic charge
ε	Dielectric constant
σ	van der Waal's radius

References

[1] M. J. Duer, *Introduction to solid-state NMR spectroscopy* (Oxford, UK, 2004)
[2] I. M. Ward, Structure and properties of oriented polymers, (Springer, 1997), p. 536
[3] H. Tang, M. H. Malakooti, H. A. Sodano, Relationship between orientation factor of lead zirconate titanate nanowires and dielectric permittivity of nanocomposites. Applied Physics Letters. 2013 Nov 25;103(22):222901.
[4] E. Zaminpayma, K. Mirabbaszadeh, Interaction between single-walled carbon nanotubes and polymers: A molecular dynamics simulation study with reactive force field. Computational Materials Science. 2012 Jun 1;58:7-11.
[5] H. Sun, S. J. Mumby, J. R. Maple, A. T. Hagler, An ab initio CFF93 all-atom force field for polycarbonates. Journal of the American Chemical society. 1994 Apr;116(7):2978-87.
